# Supplementary figures and images for: Incorporating regulatory interactions into gene-set analyses for GWAS data: A controlled analysis with the MAGMA tool
Source: PLoS Comput Biol. 2022 Mar 22;18(3):e1009908. doi: 10.1371/journal.pcbi.1009908 (PMC8939811; doi:10.1371/journal.pcbi.1009908)

(A)

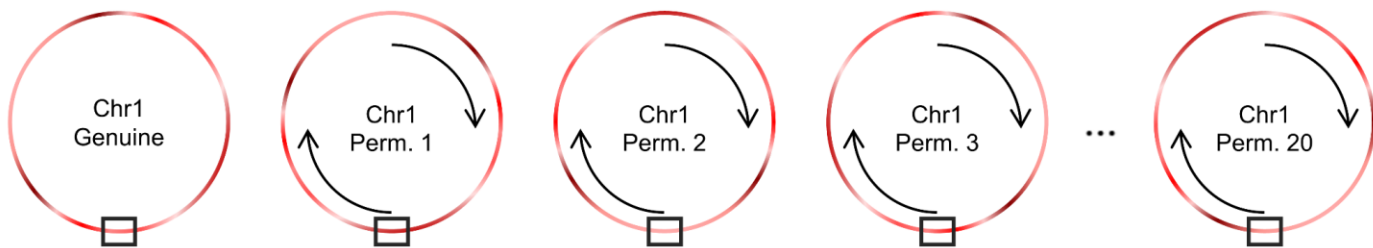

(B)

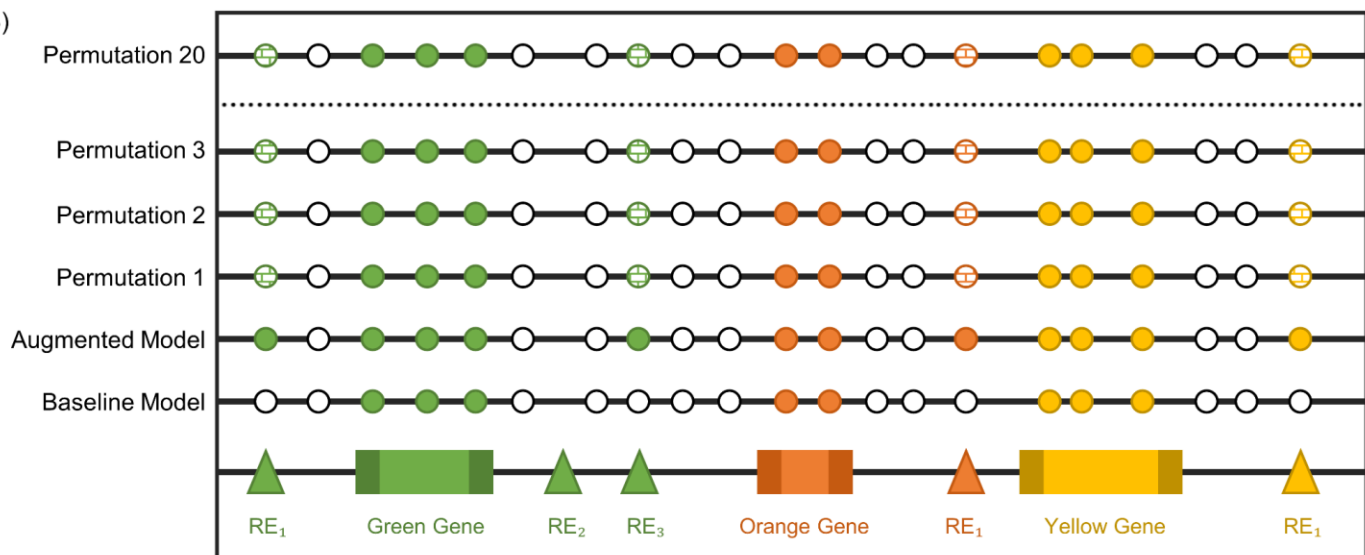

(C)

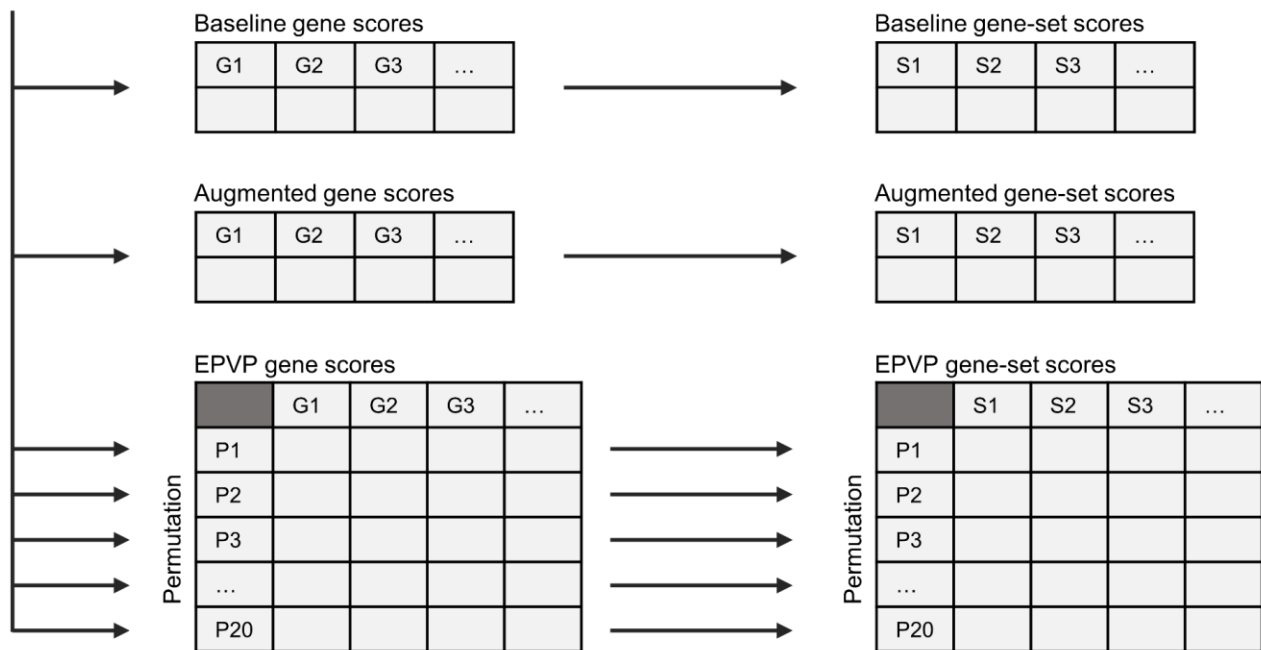

Supplement: S1 Fig — (A) SNVs in a dataset of GWAS summary statistics are arranged along their coordinate positions. For each permutation, SNV-level associations (depicted as varying tones of red) are shifted collectively by a randomly-chosen index within each chromosome (note that, only one chromosome is shown). Chromosomes are treated as circular, such that indices surpassing the end of a chromosome, are pushed back to its start. (B) A small region of our chromosome (bordered by a black box in A) is shown in detail. Genes (rectangles) and their regulatory elements (triangles) are illustrated on one track, and SNVs (circles) are repeated across multiple, separate tracks (one for each of the labelled scenarios). For each permutation of EPVP, SNVs located within regulatory elements or flanks of each gene, but not part of their baseline-model definition (that is, our so-called extragenic SNVs), are assigned background SNV-level associations (depicted as circles with a pattern-fill on runs with permuted data). SNV-level associations amongst SNVs mapped to a gene via the baseline model are left unaltered (depicted as circles with a solid, non-white fill). SNVs not mapped to any gene (according to the mapping) are also shown, but do not affect the score of any gene (depicted as circles with a solid, white fill). (C) Gene scores (for genes G1, G2, G3, …) and gene-set scores (for gene sets S1, S2, S3, …) are computed with the baseline model using unpermuted data (Baseline Model in B), with the augmented model using unpermuted data (Augmented Model in B), as well as with the augmented model using permuted data (20 independent permutations of EPVP; Permutation 1–20 in B). Row-wise evaluations focus on the numbers of significant genes or gene sets detected, whereas column-wise evaluations focus on responses of individual genes or gene sets to augmentation. Abbreviations: Chr1 (Chromosome 1); Perm (Permutation); RE (regulatory element); G1 (Gene 1); S1 (Gene Set 1); P1 (Permutation 1). (PDF) [file pcbi.1009908.s001.pdf]
