# Supplementary material for: Incorporating regulatory interactions into gene-set analyses for GWAS data: A controlled analysis with the MAGMA tool
Source: PLoS Comput Biol. 2022 Mar 22;18(3):e1009908. doi: 10.1371/journal.pcbi.1009908 (PMC8939811; doi:10.1371/journal.pcbi.1009908)
Supplement: S2 Fig — If the size of genes is unrelated to their tendency to be significant, the cumulative distribution of significant genes ranked by their size is expected to fall approximately on the diagonal. Downward deviation from the diagonal implies that large genes are preferentially significant, while upward deviation from the diagonal implies that small genes are preferentially significant. A deviation from the diagonal is not necessarily a bias, because genes associated with a phenotype might have a genuine tendency to be either large or small (after all, immune-related genes tend to be small and neuron-related genes tend to be large [89]). To evaluate whether or not augmentation results in a confounding gene-size effect (that is, a systematic effect on gene scores that occurs with random augmentation), it is therefore necessary to test for a consistent deviation (that is, consistent across phenotypes and in terms of directionality) from the diagonal that is induced (that is, occurs relative the baseline-model distribution) by random augmentation. We expect at least that the baseline-model distribution does not show evidence for a gene-size effect, since MAGMA’s gene scores are said to be well controlled for gene size (see original publication [31] and MAGMA website for details). (A) Hypothetical illustration of a gene-size effect where larger genes are preferentially significant not just with genuine augmentation (green), but also with matched, random augmentation (grey; one distribution for each of 20 independent permutations of EPVP). Except for the baseline-model distribution (blue), these distributions are not based on real data and serve only to clarify the previous points. We point out that, as expected, no gene-size effect is observed with the baseline model. In most cases involving real data (B-E, G, H), there was no prominent (that is, observable) relationship between the size of genes and their tendency to be significant. (F) However, augmentation with random coun [file pcbi.1009908.s002.pdf]

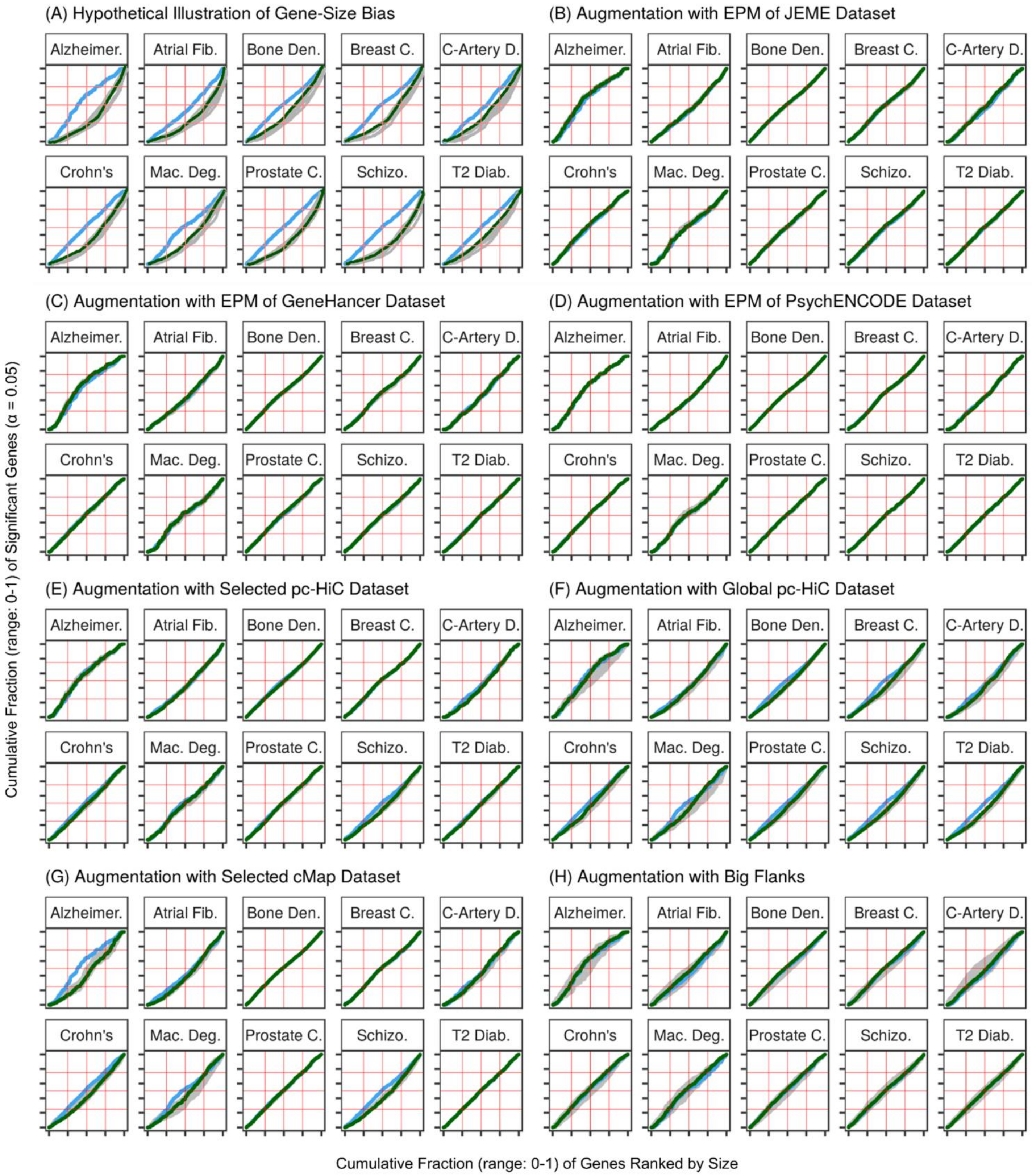
(A) Hypothetical Illustration of Gene-Size Bias
(B) Augmentation with EPM of JEME Dataset
(C) Augmentation with EPM of GeneHancer Dataset
(D) Augmentation with EPM of PsychENCODE Dataset
(E) Augmentation with Selected pc-HiC Dataset
(F) Augmentation with Global pc-HiC Dataset
(G) Augmentation with Selected cMap Dataset
(H) Augmentation with Big Flanks
Alzheimer.
Atrial Fib.
Bone Den.
Breast C.
C-Artery D.
Crohn's
Mac. Deg.
Prostate C.
Schizo.
T2 Diab.
Cumulative Fraction (range: 0-1) of Significant Genes (α = 0.05)
Cumulative Fraction (range: 0-1) of Genes Ranked by Size
