# Supplementary material for: Incorporating regulatory interactions into gene-set analyses for GWAS data: A controlled analysis with the MAGMA tool
Source: PLoS Comput Biol. 2022 Mar 22;18(3):e1009908. doi: 10.1371/journal.pcbi.1009908 (PMC8939811; doi:10.1371/journal.pcbi.1009908)
Supplement: S3 Fig — The following figures are enlargements of (A) S2B Fig and (B) S2F Fig, respectively (refer to the caption of S2 Fig for an explanation). (PDF) [file pcbi.1009908.s003.pdf]

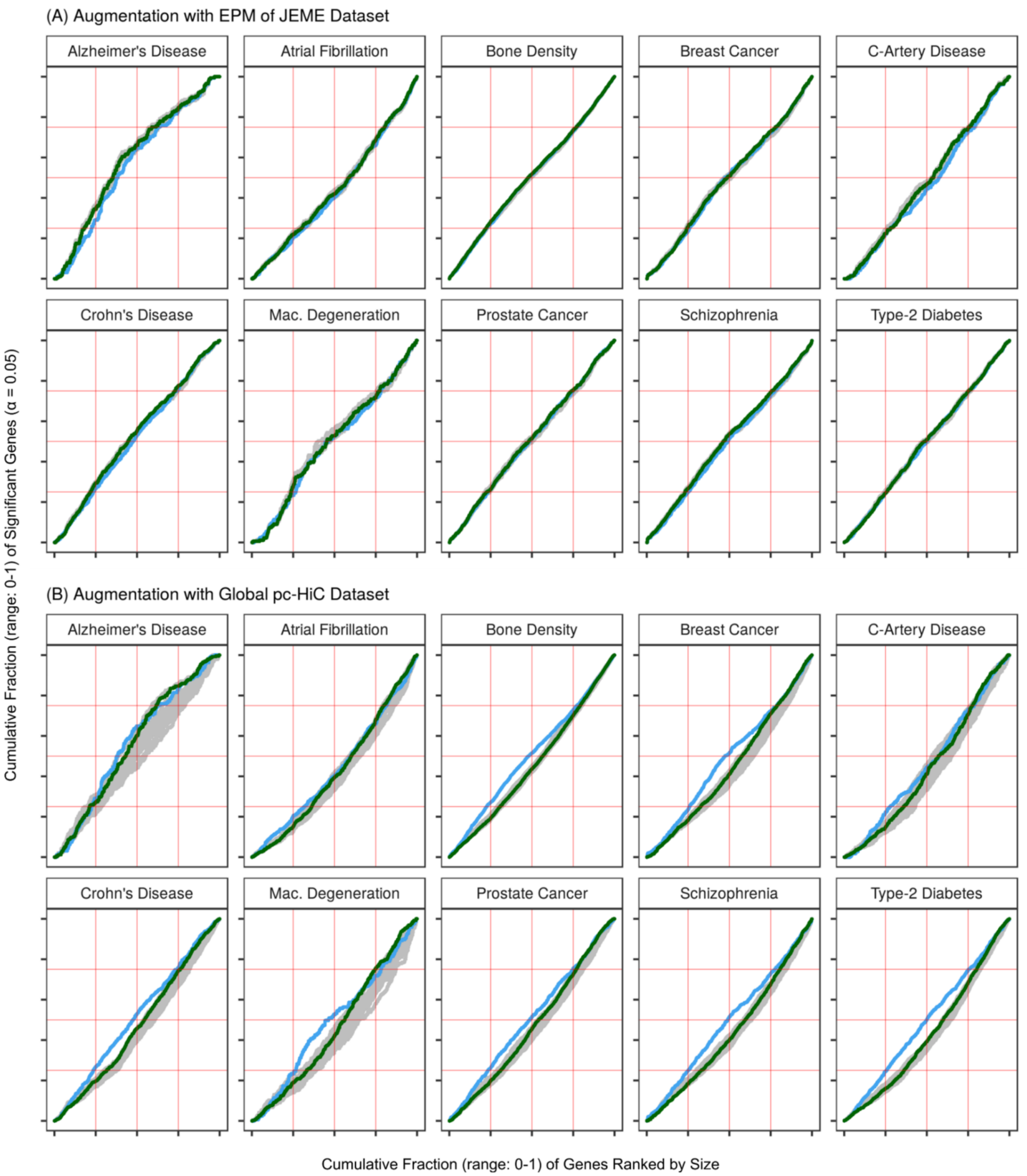
(A) Augmentation with EPM of JEME Dataset
Alzheimer's Disease
Atrial Fibrillation
Bone Density
Breast Cancer
C-Artery Disease
Crohn's Disease
Mac. Degeneration
Prostate Cancer
Schizophrenia
Type-2 Diabetes
(B) Augmentation with Global pc-HiC Dataset
Alzheimer's Disease
Atrial Fibrillation
Bone Density
Breast Cancer
C-Artery Disease
Crohn's Disease
Mac. Degeneration
Prostate Cancer
Schizophrenia
Type-2 Diabetes
Cumulative Fraction (range: 0-1) of Significant Genes (α = 0.05)
Cumulative Fraction (range: 0-1) of Genes Ranked by Size
