# Supplementary material for: Incorporating regulatory interactions into gene-set analyses for GWAS data: A controlled analysis with the MAGMA tool
Source: PLoS Comput Biol. 2022 Mar 22;18(3):e1009908. doi: 10.1371/journal.pcbi.1009908 (PMC8939811; doi:10.1371/journal.pcbi.1009908)
Supplement: S4 Fig — This is an extension of Fig 6 from the Main Text to all other phenotypes (refer to the caption of Fig 6 for an explanation). (A) Alzheimer’s Disease, (B) Bone Density, (C) Breast Cancer, (D) C-Artery Disease (Coronary-Artery Disease), (E) Crohn’s Disease, (F) Mac. Degeneration (Macular Degeneration), (G) Prostate Cancer, (H) Schizophrenia, and (I) Type-2 Diabetes. (PDF) [file pcbi.1009908.s004.pdf]

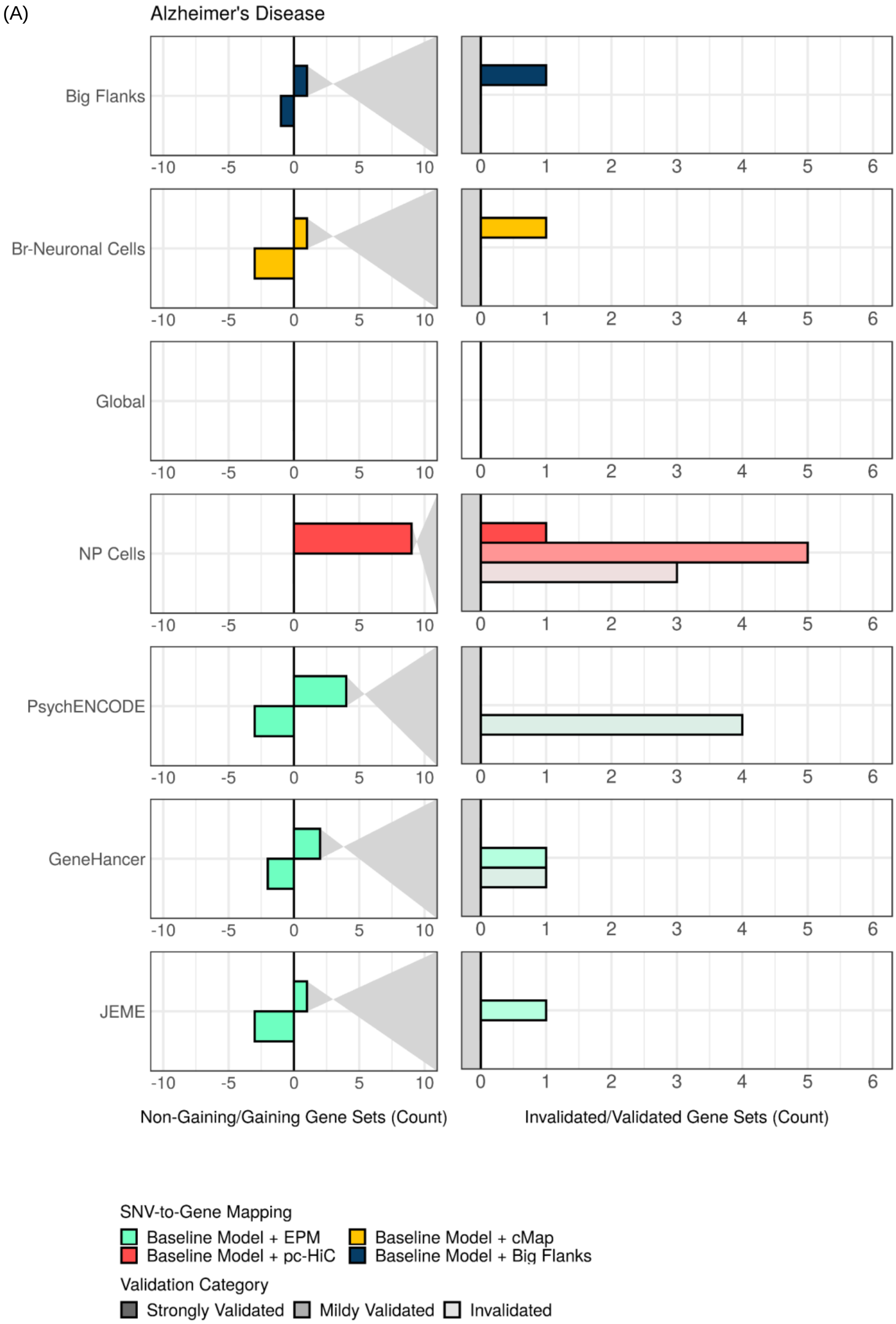
(A)
Alzheimer's Disease
Big Flanks
Br-Neuronal Cells
Global
NP Cells
PsychENCODE
GeneHancer
JEME
Non-Gaining/Gaining Gene Sets (Count)
Invalidated/Validated Gene Sets (Count)
SNV-to-Gene Mapping
Baseline Model + EPM
Baseline Model + cMap
Baseline Model + pc-HiC
Baseline Model + Big Flanks
Validation Category
Strongly Validated
Mildy Validated
Invalidated

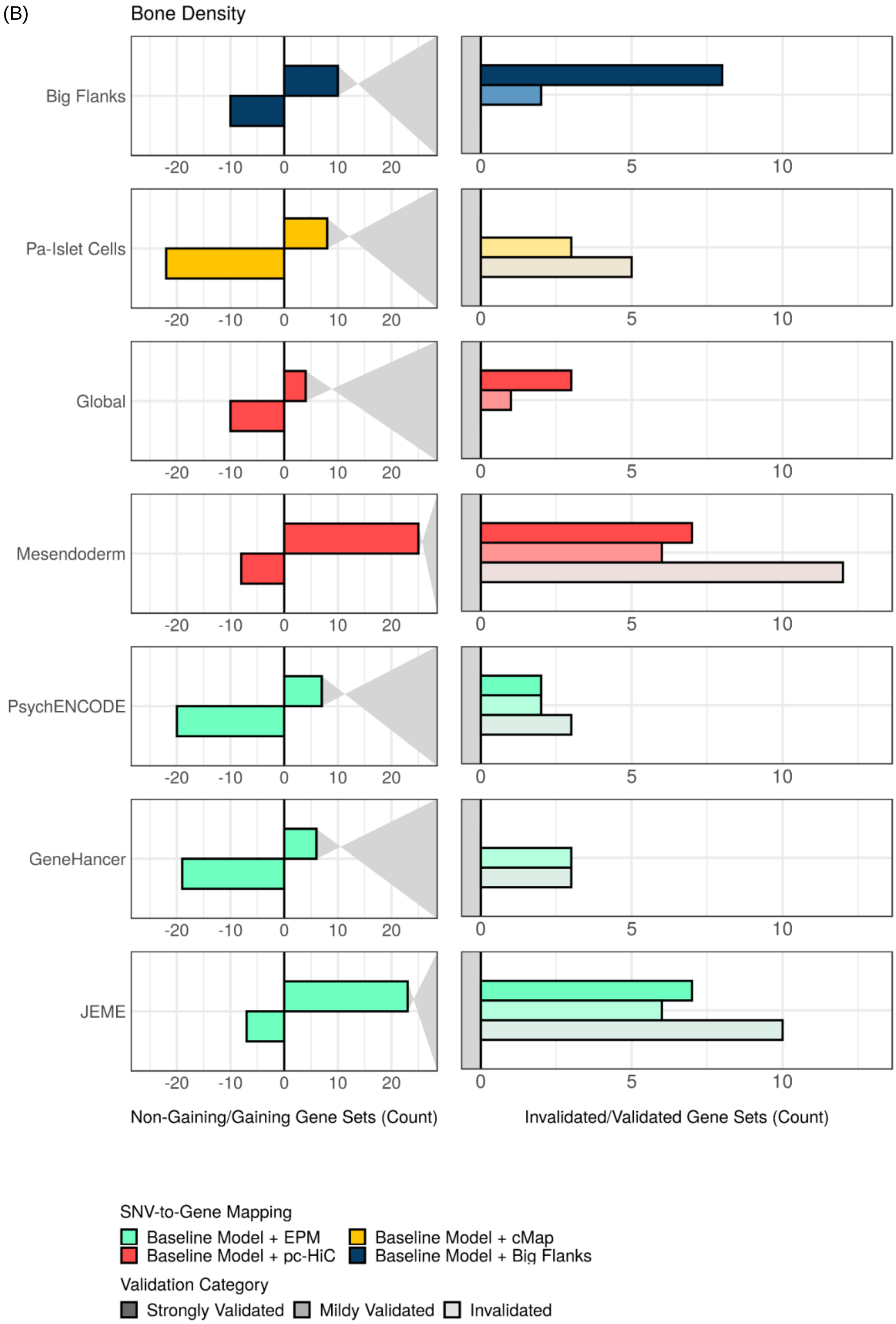

(B)
Bone Density
Big Flanks
Pa-Islet Cells
Global
Mesendoderm
PsychENCODE
GeneHancer
JEME
-20
-10
0
10
20
5
Non-Gaining/Gaining Gene Sets (Count)
Invalidated/Validated Gene Sets (Count)
SNV-to-Gene Mapping
Baseline Model + EPM
Baseline Model + cMap
Baseline Model + pc-HiC
Baseline Model + Big Flanks
Validation Category
Strongly Validated
Mildy Validated
Invalidated

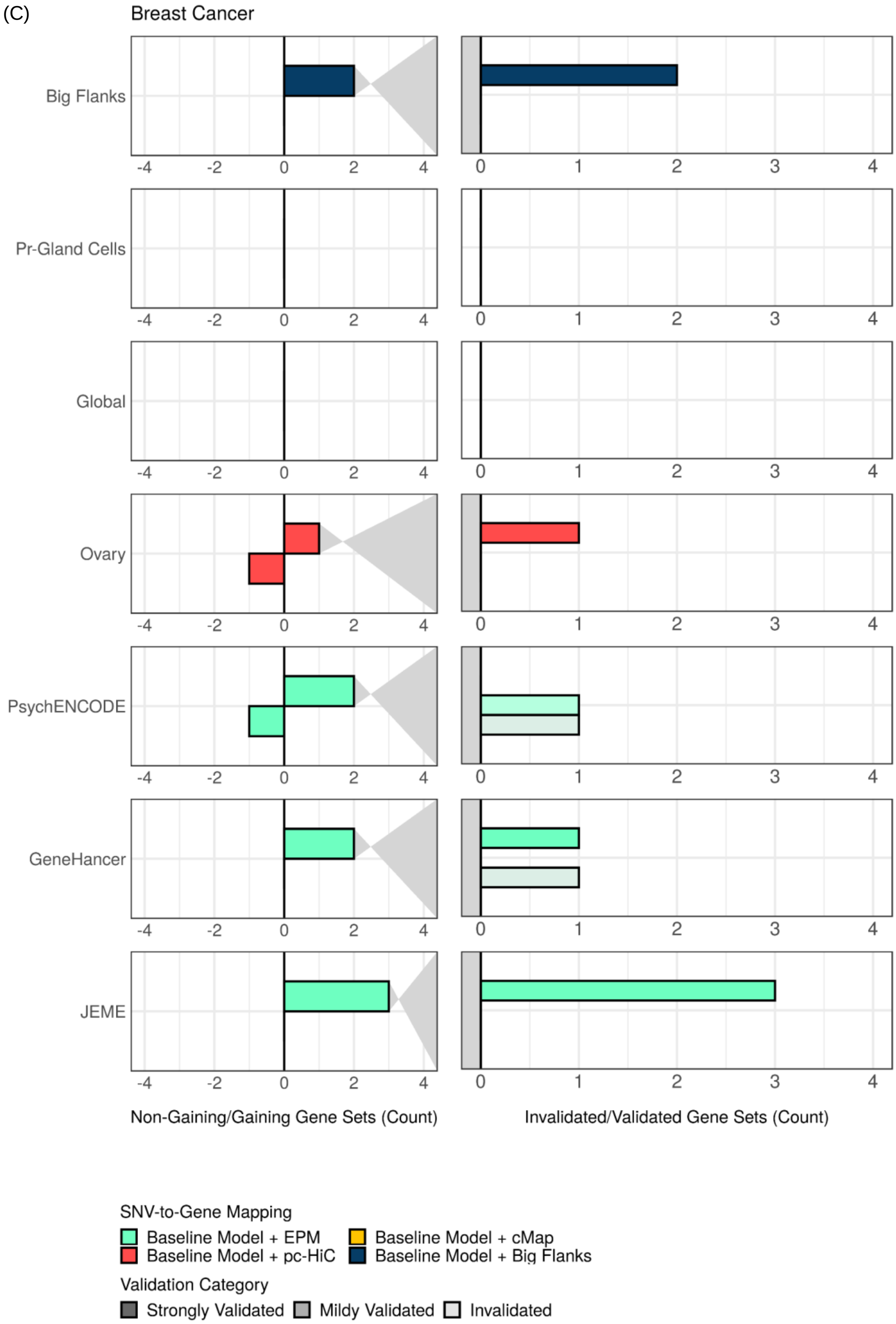
(C)
Breast Cancer
Big Flanks
Pr-Gland Cells
Global
Ovary
PsychENCODE
GeneHancer
JEME
-4 -2 0 2 4
0 1 2 3 4
Non-Gaining/Gaining Gene Sets (Count)
Invalidated/Validated Gene Sets (Count)
SNV-to-Gene Mapping
Baseline Model + EPM
Baseline Model + pc-HiC
Baseline Model + cMap
Baseline Model + Big Flanks
Validation Category
Strongly Validated
Mildy Validated
Invalidated

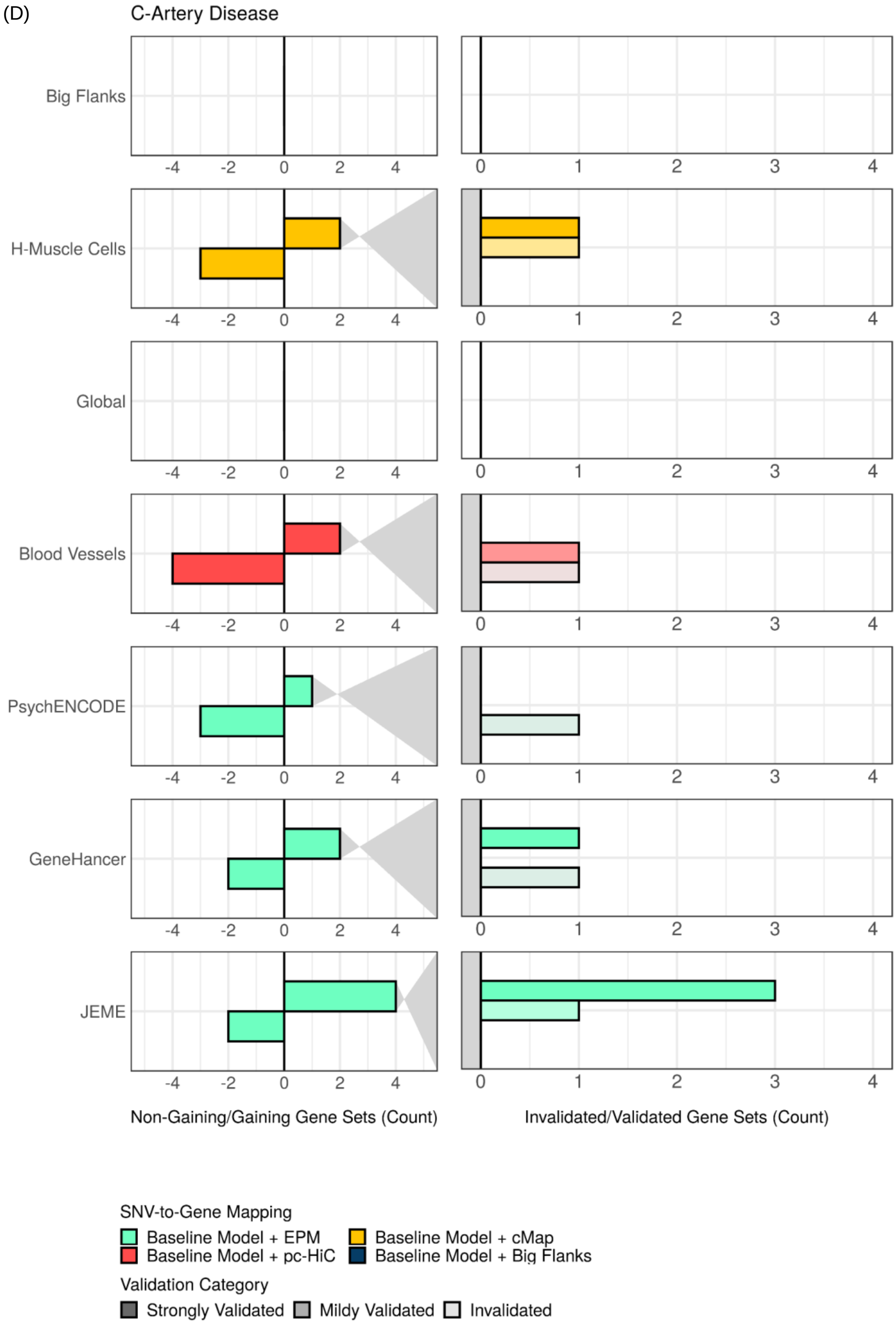

(D)
C-Artery Disease
Big Flanks
H-Muscle Cells
Global
Blood Vessels
PsychENCODE
GeneHancer
JEME
-4
-2
0
2
4
0
1
2
3
4
Non-Gaining/Gaining Gene Sets (Count)
Invalidated/Validated Gene Sets (Count)
SNV-to-Gene Mapping
Baseline Model + EPM
Baseline Model + cMap
Baseline Model + pc-HiC
Baseline Model + Big Flanks
Validation Category
Strongly Validated
Mildy Validated
Invalidated

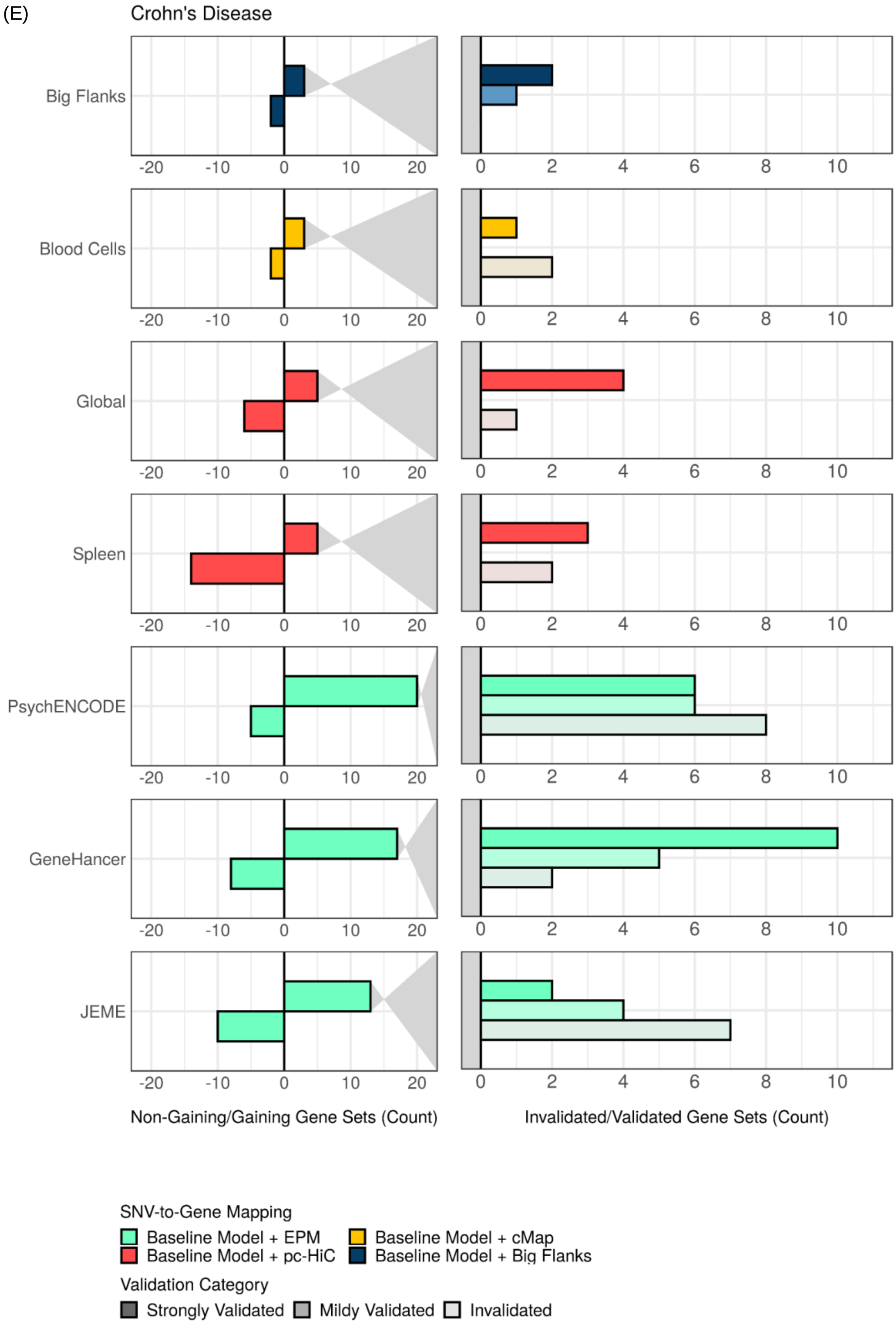
(E)
Crohn's Disease
Big Flanks
Blood Cells
Global
Spleen
PsychENCODE
GeneHancer
JEME
-20
-10
0
10
20
2
4
6
8
Non-Gaining/Gaining Gene Sets (Count)
Invalidated/Validated Gene Sets (Count)
SNV-to-Gene Mapping
Baseline Model + EPM
Baseline Model + pc-HiC
Baseline Model + cMap
Baseline Model + Big Flanks
Validation Category
Strongly Validated
Mildy Validated
Invalidated

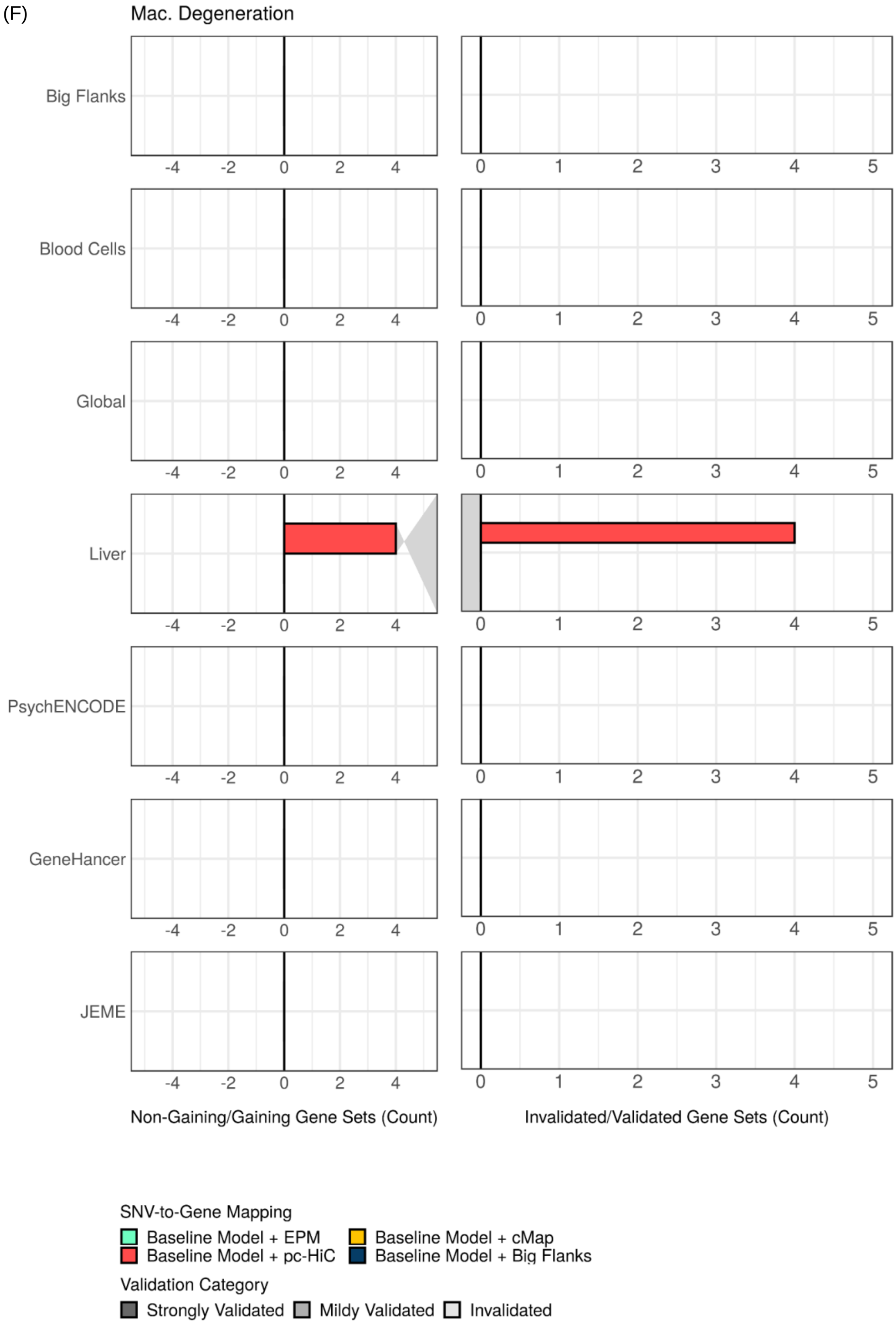

(F)
Mac. Degeneration
Big Flanks
Blood Cells
Global
Liver
PsychENCODE
GeneHancer
JEME
-4
-2
0
2
4
0
1
2
3
4
5
Non-Gaining/Gaining Gene Sets (Count)
Invalidated/Validated Gene Sets (Count)
SNV-to-Gene Mapping
Baseline Model + EPM
Baseline Model + cMap
Baseline Model + pc-HiC
Baseline Model + Big Flanks
Validation Category
Strongly Validated
Mildy Validated
Invalidated

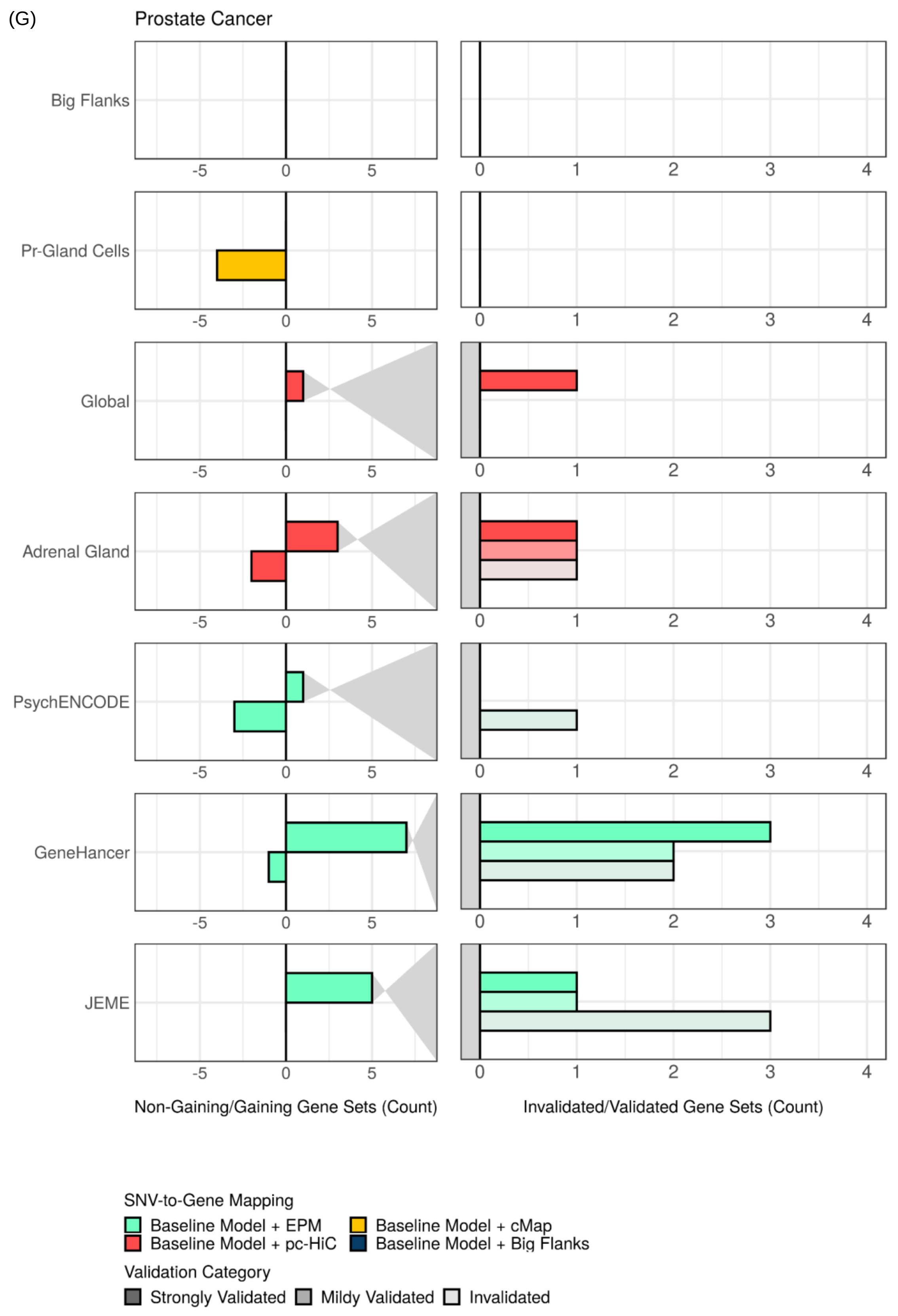

(G)
Prostate Cancer
Big Flanks
Pr-Gland Cells
Global
Adrenal Gland
PsychENCODE
GeneHancer
JEME
-5
0
5
1
2
3
4
Non-Gaining/Gaining Gene Sets (Count)
Invalidated/Validated Gene Sets (Count)
SNV-to-Gene Mapping
Baseline Model + EPM
Baseline Model + pc-HiC
Baseline Model + cMap
Baseline Model + Big Flanks
Validation Category
Strongly Validated
Mildy Validated
Invalidated

(H)

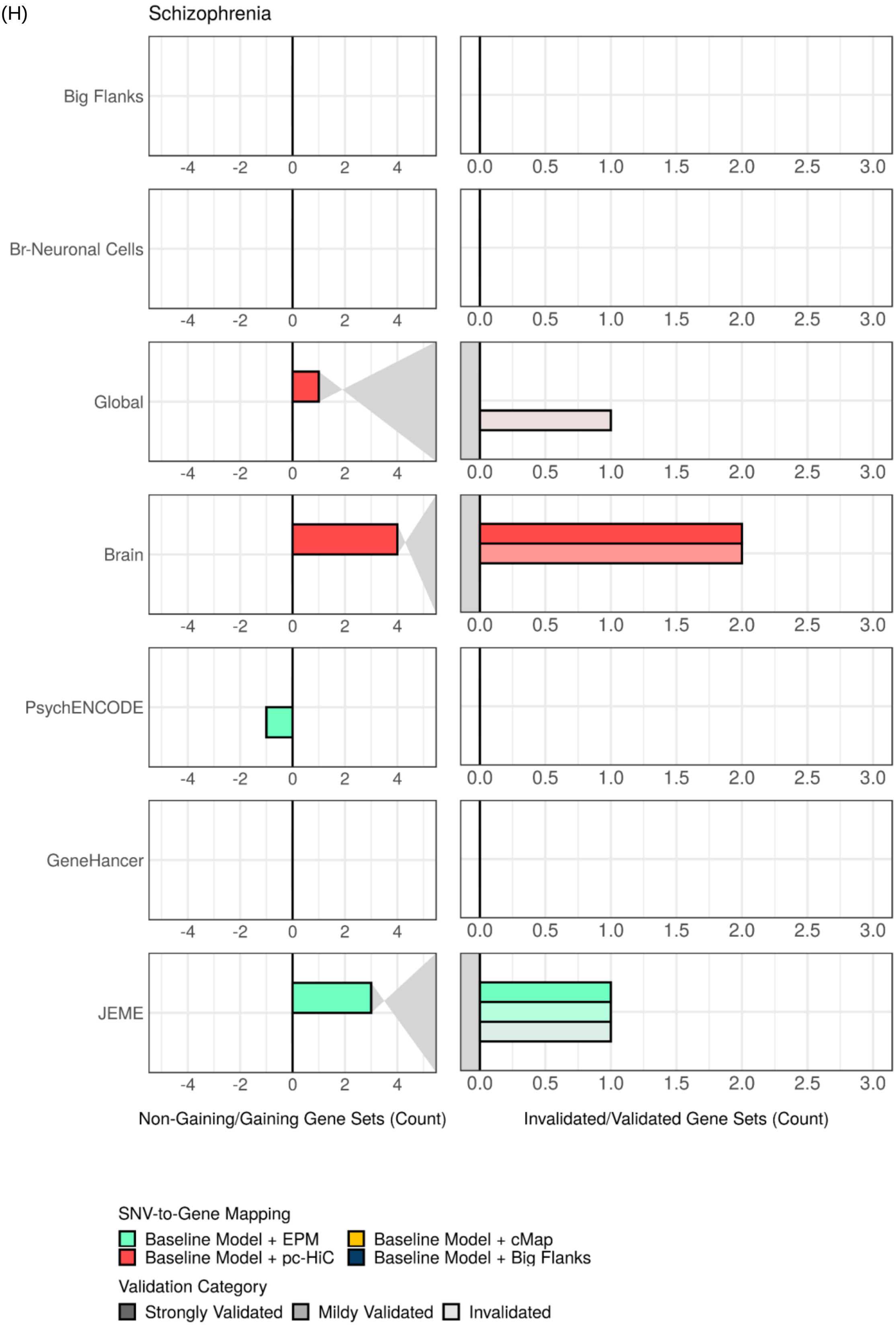
Schizophrenia
Big Flanks
Br-Neuronal Cells
Global
Brain
PsychENCODE
GeneHancer
JEME
-4
-2
0
2
4
0.0
0.5
1.0
1.5
2.0
2.5
3.0
Non-Gaining/Gaining Gene Sets (Count)
Invalidated/Validated Gene Sets (Count)
SNV-to-Gene Mapping
Baseline Model + EPM
Baseline Model + pc-HiC
Baseline Model + cMap
Baseline Model + Big Flanks
Validation Category
Strongly Validated
Mildy Validated
Invalidated

(I)

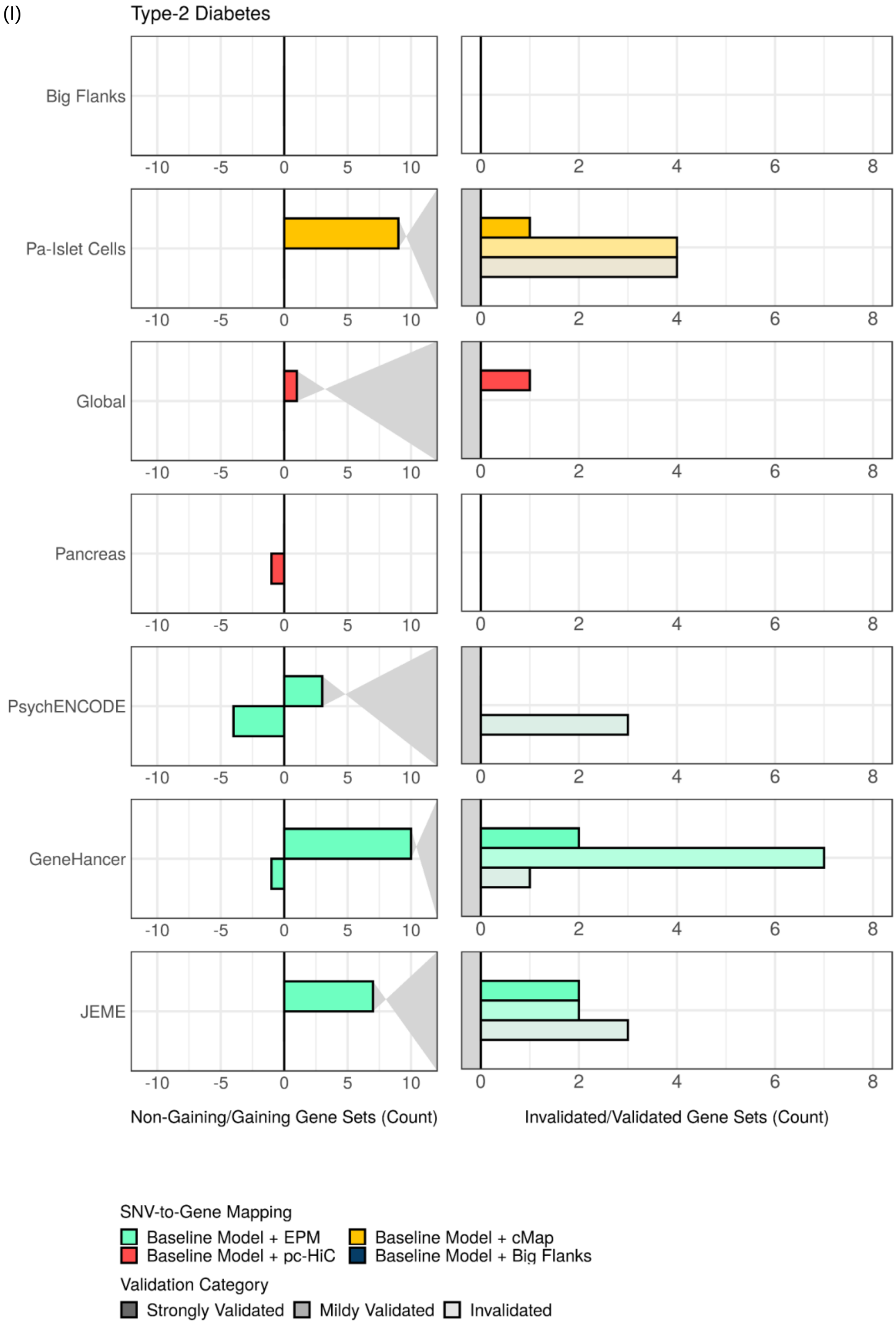

Type-2 Diabetes
Big Flanks
Pa-Islet Cells
Global
Pancreas
PsychENCODE
GeneHancer
JEME
-10
-5
0
5
10
2
4
6
8
Non-Gaining/Gaining Gene Sets (Count)
Invalidated/Validated Gene Sets (Count)
SNV-to-Gene Mapping
Baseline Model + EPM
Baseline Model + cMap
Baseline Model + pc-HiC
Baseline Model + Big Flanks
Validation Category
Strongly Validated
Mildy Validated
Invalidated
