# Supplementary material for: Incorporating regulatory interactions into gene-set analyses for GWAS data: A controlled analysis with the MAGMA tool
Source: PLoS Comput Biol. 2022 Mar 22;18(3):e1009908. doi: 10.1371/journal.pcbi.1009908 (PMC8939811; doi:10.1371/journal.pcbi.1009908)

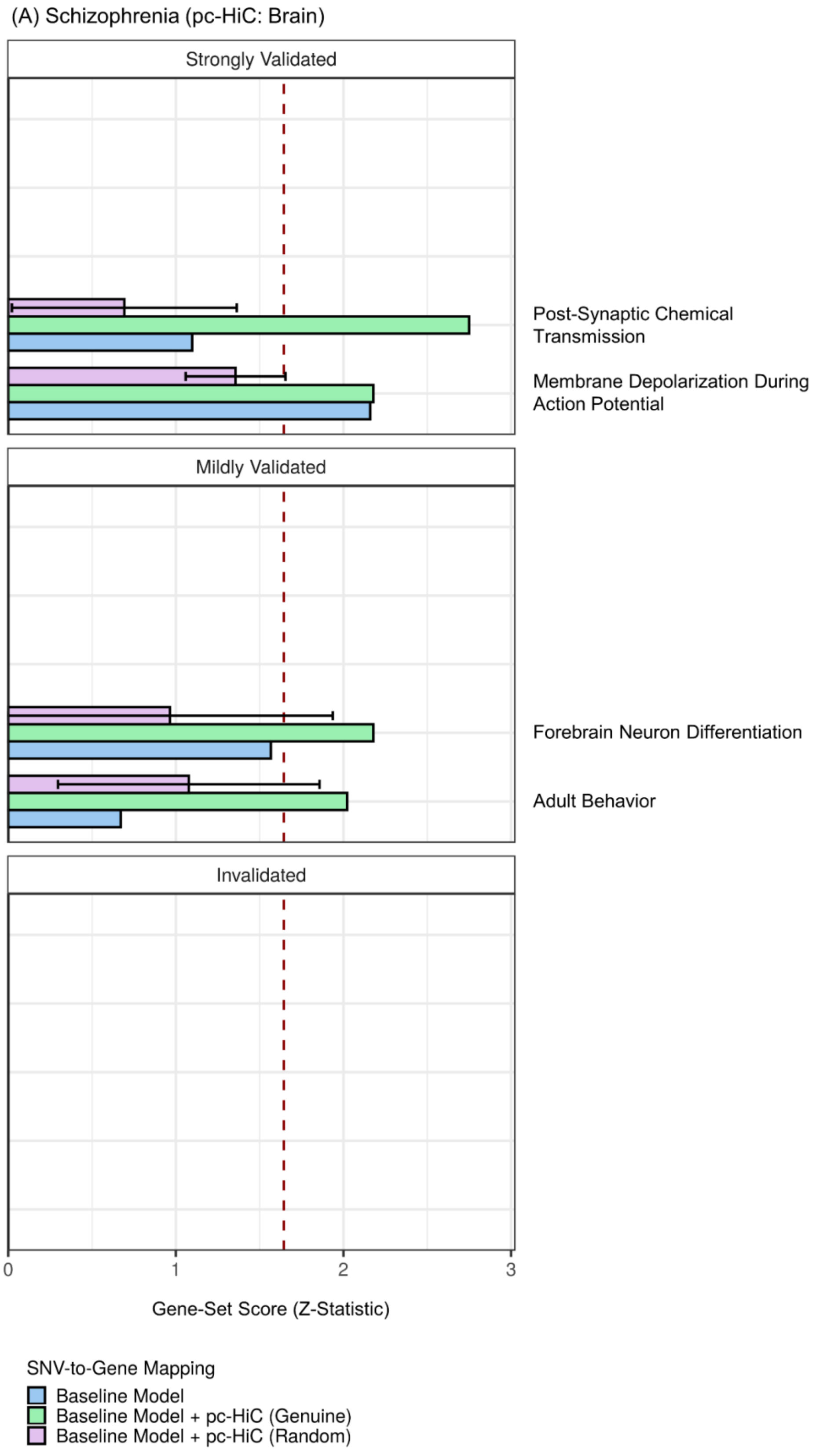
(A) Schizophrenia (pc-HiC: Brain)
Strongly Validated
Post-Synaptic Chemical Transmission
Membrane Depolarization During Action Potential
Mildly Validated
Forebrain Neuron Differentiation
Adult Behavior
Invalidated
0
1
2
3
Gene-Set Score (Z-Statistic)
SNV-to-Gene Mapping
Baseline Model
Baseline Model + pc-HiC (Genuine)
Baseline Model + pc-HiC (Random)

(B) Schizophrenia

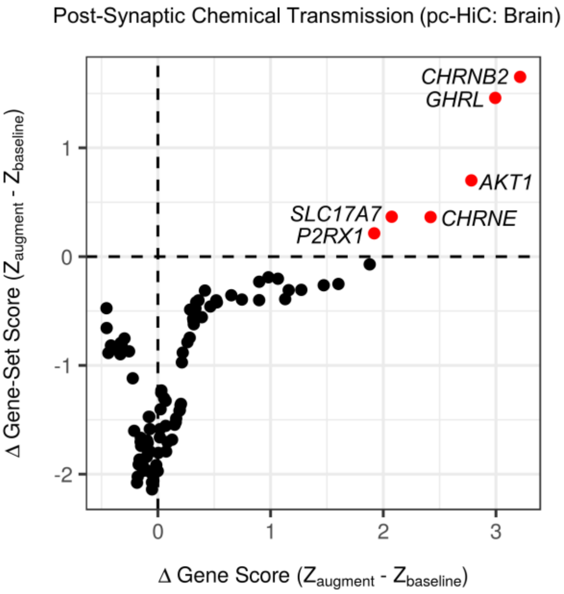

Supplement: S5 Fig — (A) A comparison between gene-set scores (that is, each score is based on the probit transformation of one minus the relevant, FDR-adjusted, upper-tail p-value) obtained using the baseline model, the baseline model augmented with genuine regulatory interactions, and the baseline model augmented with matched, random regulatory interactions. Bigger, positive scores imply stronger enrichment for phenotype association. For random augmentation, counts and error bars represent the mean and standard deviation, respectively (based on 20 independent permutations of EPVP). Only gene sets detected as statistically significant with genuine augmentation are shown. Red, dashed line shows the significance cut-off (α = 0.05). Each gene set was assigned to one of three validation categories to reflect how pronounced a gain was with genuine augmentation over that with matched, random augmentation (see Main Text). (B) The gain for the post-synaptic chemical transmission gene set was robust (refer to the caption of Fig 7 and the Main Text for an explanation). Top-gaining genes that had to be removed from the gene set for its gain to be lost are labelled. (PDF) [file pcbi.1009908.s005.pdf]
