# Supplementary material for: Incorporating regulatory interactions into gene-set analyses for GWAS data: A controlled analysis with the MAGMA tool
Source: PLoS Comput Biol. 2022 Mar 22;18(3):e1009908. doi: 10.1371/journal.pcbi.1009908 (PMC8939811; doi:10.1371/journal.pcbi.1009908)

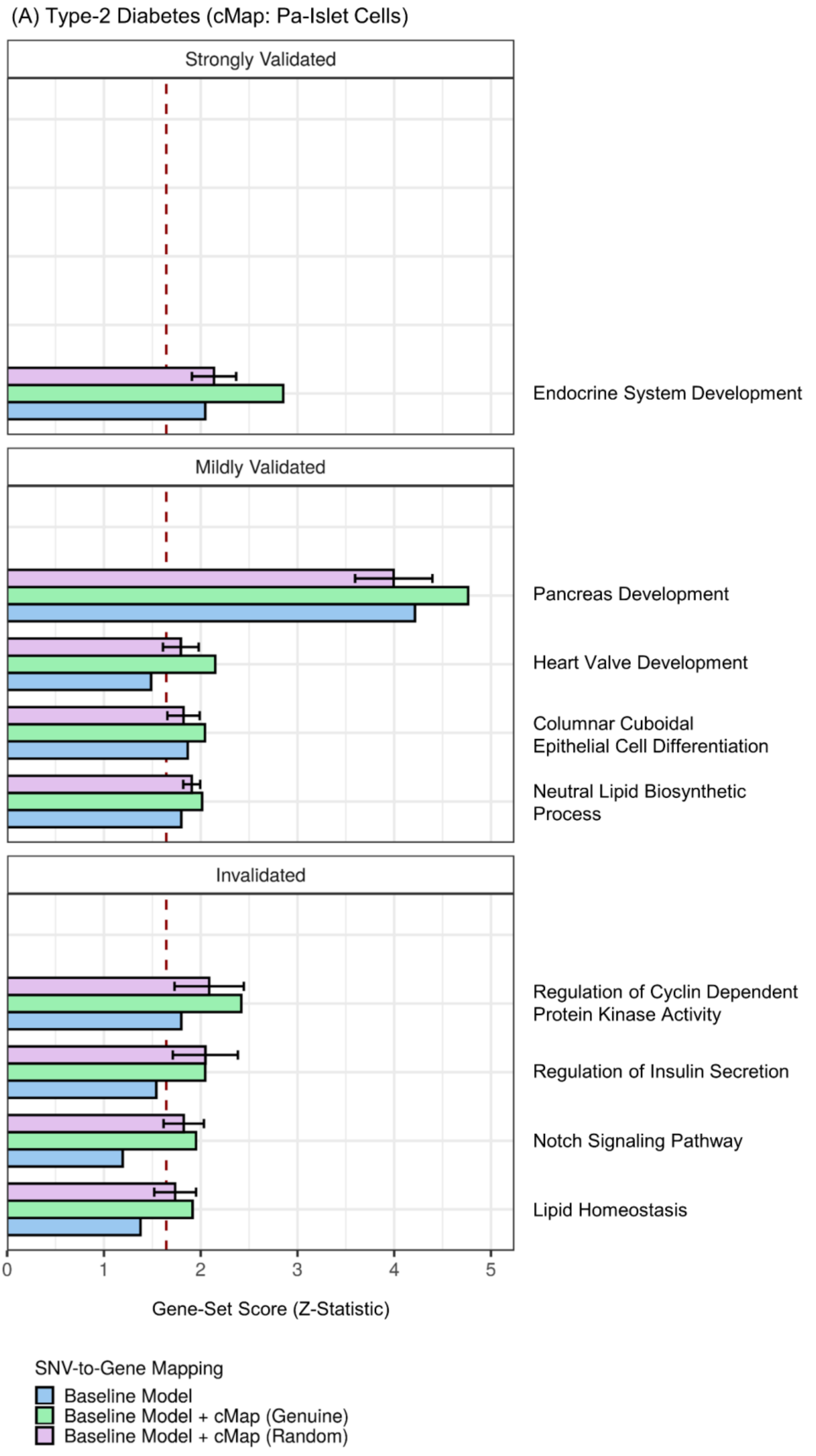
(A) Type-2 Diabetes (cMap: Pa-Islet Cells)
Strongly Validated
Endocrine System Development
Mildly Validated
Pancreas Development
Heart Valve Development
Columnar Cuboidal Epithelial Cell Differentiation
Neutral Lipid Biosynthetic Process
Invalidated
Regulation of Cyclin Dependent Protein Kinase Activity
Regulation of Insulin Secretion
Notch Signaling Pathway
Lipid Homeostasis
0
1
2
3
4
5
Gene-Set Score (Z-Statistic)
SNV-to-Gene Mapping
Baseline Model
Baseline Model + cMap (Genuine)
Baseline Model + cMap (Random)

(B) Type-2 Diabetes

Endocrine System Development (cMap: Pa-Islet Cells)

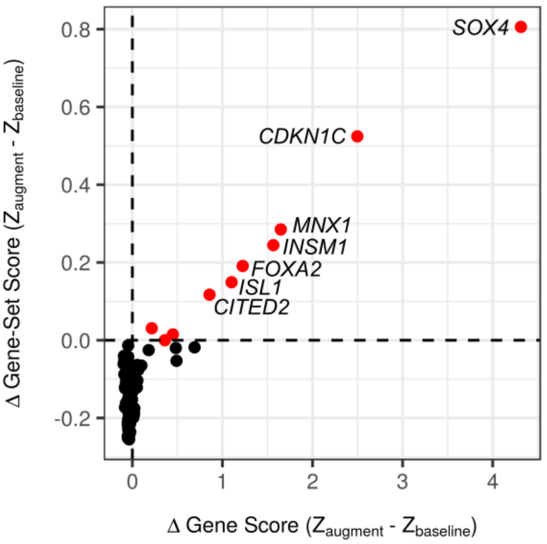

Supplement: S6 Fig — (A) A comparison between gene-set scores (that is, each score is based on the probit transformation of one minus the relevant, FDR-adjusted, upper-tail p-value) obtained using the baseline model, the baseline model augmented with genuine regulatory interactions, and the baseline model augmented with matched, random regulatory interactions (refer to the caption of S5A Fig for an explanation). (B) The gain for the endocrine system development gene set was robust (refer to the caption of Fig 7 and the Main Text for an explanation). Top-gaining genes that had to be removed from the gene set for its gain to be lost are labelled. Mapping abbreviations: Pa-Islet Cells (pancreatic-islet cells). (PDF) [file pcbi.1009908.s006.pdf]
