# Supplementary material for: Incorporating regulatory interactions into gene-set analyses for GWAS data: A controlled analysis with the MAGMA tool
Source: PLoS Comput Biol. 2022 Mar 22;18(3):e1009908. doi: 10.1371/journal.pcbi.1009908 (PMC8939811; doi:10.1371/journal.pcbi.1009908)

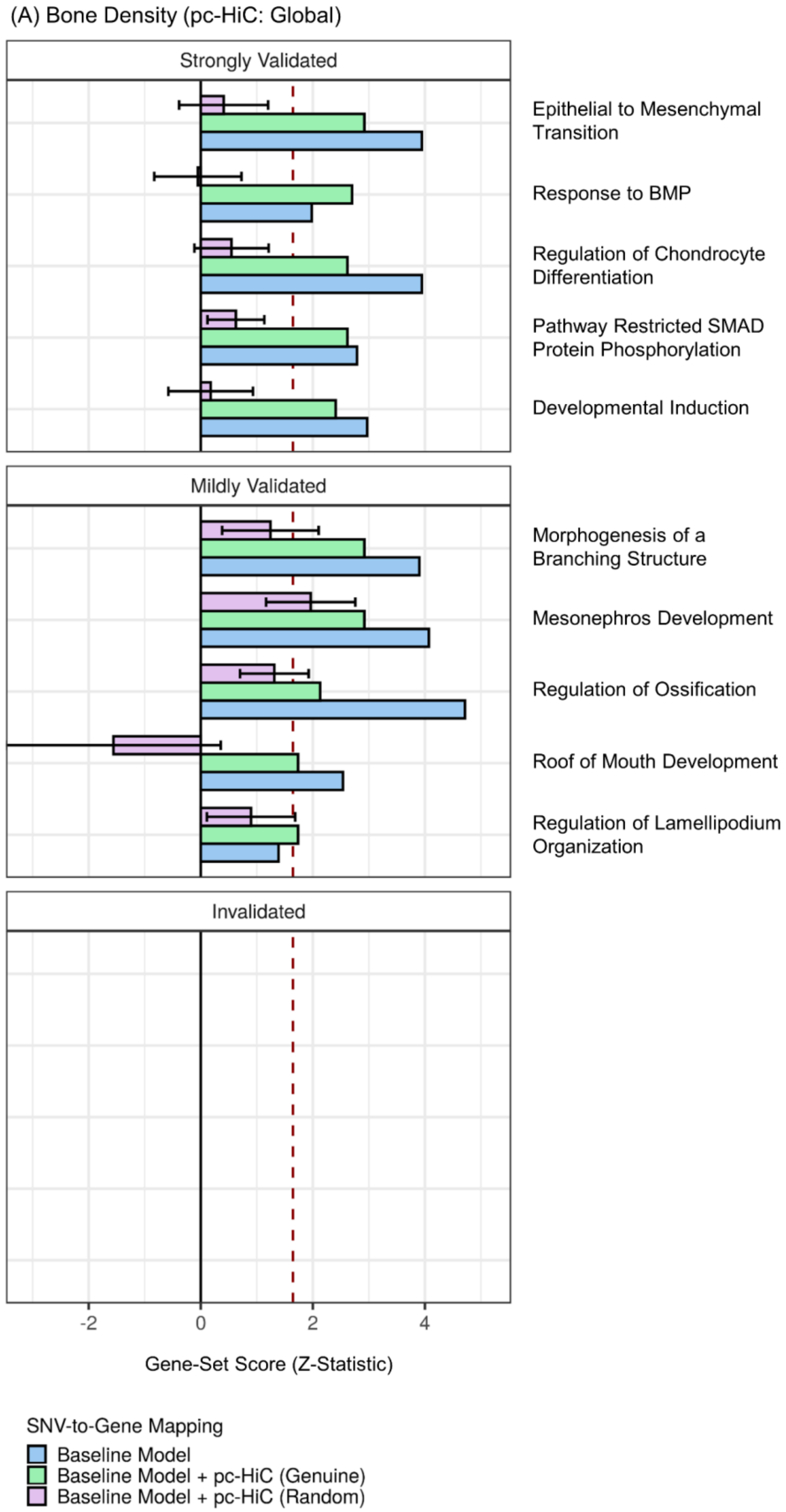

(A) Bone Density (pc-HiC: Global)
Strongly Validated
Epithelial to Mesenchymal Transition
Response to BMP
Regulation of Chondrocyte Differentiation
Pathway Restricted SMAD Protein Phosphorylation
Developmental Induction
Mildly Validated
Morphogenesis of a Branching Structure
Mesonephros Development
Regulation of Ossification
Roof of Mouth Development
Regulation of Lamellipodium Organization
Invalidated
-2
0
2
4
Gene-Set Score (Z-Statistic)
SNV-to-Gene Mapping
Baseline Model
Baseline Model + pc-HiC (Genuine)
Baseline Model + pc-HiC (Random)

(B) Bone Density

Response to BMP (pc-HiC: Global)

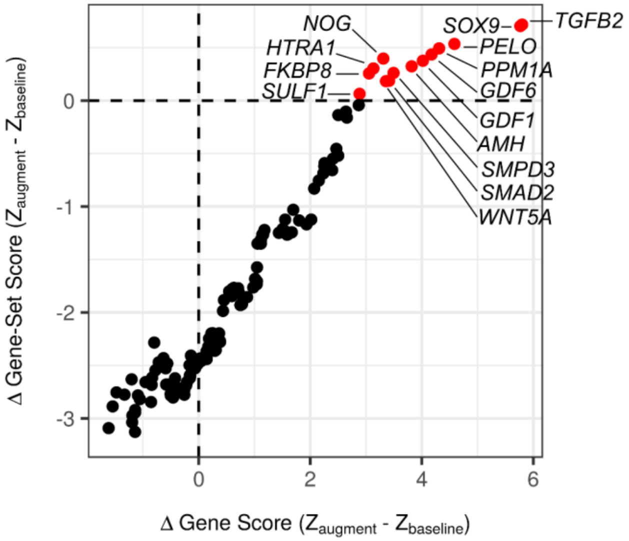

(C) Bone Density

Response to BMP (pc-HiC: Global)

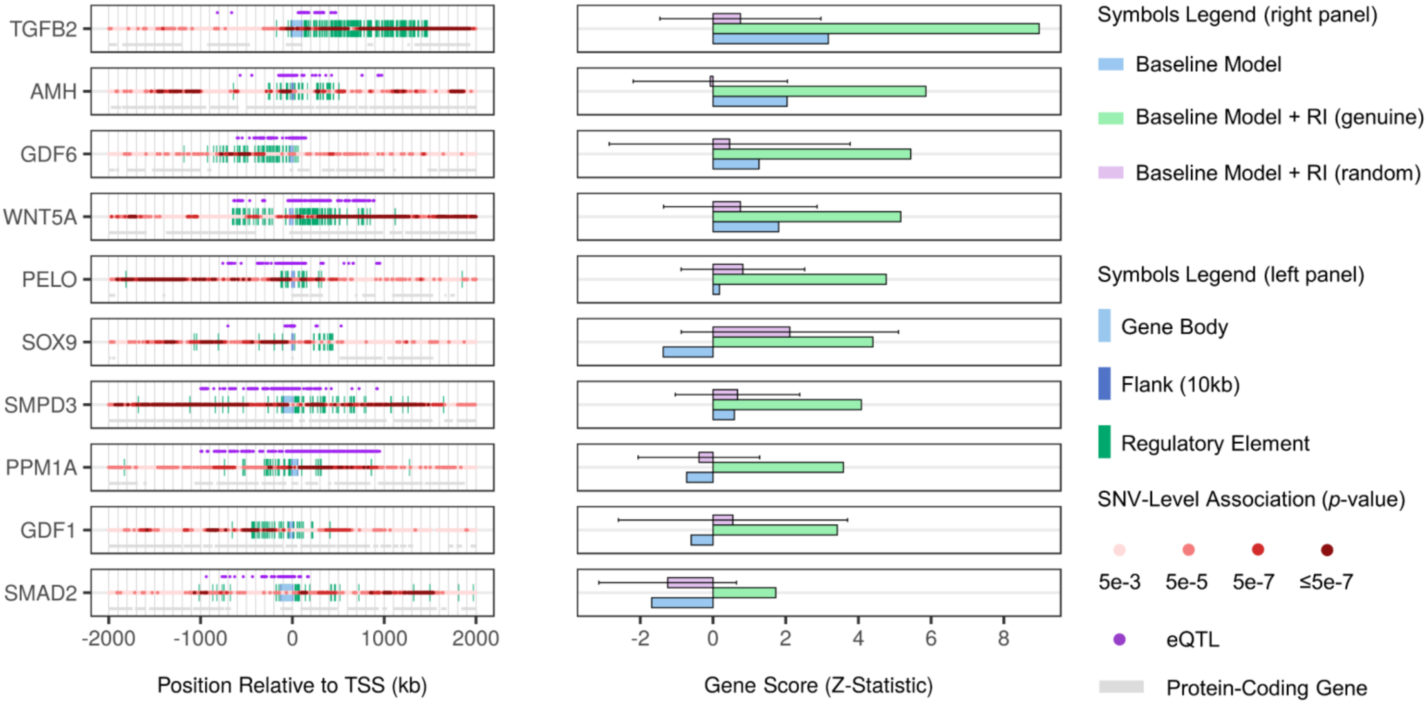

Supplement: S7 Fig — (A) A comparison between gene-set scores (that is, each score is based on the probit transformation of one minus the relevant, FDR-adjusted, upper-tail p-value) obtained using the baseline model, the baseline model augmented with genuine regulatory interactions, and the baseline model augmented with matched, random regulatory interactions (refer to the caption of S5A Fig for an explanation). To save space, only a maximum of five gene sets per validation category are shown (selected according to their gene-set score with genuine augmentation). Overall, augmentation of the baseline model with the global pc-HiC dataset of regulatory interactions, led to the detection of 14 gene sets significantly associated with bone density. Only four of these gene sets gained from augmentation, with three of the gains being strongly validated (response to BMP, negative regulation of small GTPase mediated signal transduction, and mesenchymal cell proliferation) and one mildly validated (regulation of lamellipodium organization) by the EPVP procedure. (B) The strongly validated gain for the gene set, response to BMP (officially, go_response_to_BMP), which is relevant to bone formation [90] and contained 171 genes (note that, 153 of these genes had a score with both models, and that the other 18 genes did not have a score with either model), was the only robust gain (refer to the caption of Fig 7 and to the Main Text for an explanation) (S11 Table). More precisely, the gain for this gene set was lost only after removal of the 14 top-gaining genes from the gene set (namely and in order, TGFB2, SOX9, PELO, PPM1A, GDF6, GDF1, AMH, SMPD3, SMAD2, WNT5A, NOG, HTRA1, FKBP8, and SULF1). (C) For many of the genes mentioned, we were able to identify interesting regulatory elements located in bone-density associated loci, and to additionally support their link to the relevant genes with eQTL data (refer to the caption of Fig 8 for an explanation). For example, AMH gained from eQTL-supported regula [file pcbi.1009908.s007.pdf]
