# Supplementary material for: Incorporating regulatory interactions into gene-set analyses for GWAS data: A controlled analysis with the MAGMA tool
Source: PLoS Comput Biol. 2022 Mar 22;18(3):e1009908. doi: 10.1371/journal.pcbi.1009908 (PMC8939811; doi:10.1371/journal.pcbi.1009908)
Supplement: S8 Fig — (A) A comparison between gene-set scores (that is, each score is based on the probit transformation of one minus the relevant, FDR-adjusted, upper-tail p-value) obtained using the baseline model, the baseline model augmented with genuine regulatory interactions, and the baseline model augmented with matched, random regulatory interactions (refer to the caption of S5A Fig for an explanation). Overall, augmentation of the baseline model with the EPM of GeneHancer dataset of regulatory interactions, led to the detection of eight gene sets significantly associated with prostate cancer. Seven of these gene sets gained from augmentation, and amongst them, three gains were strongly validated, two gains were mildly validated, and two gains were invalidated, by the EPVP procedure. (B) Gains were robust only for the three strongly validated gene sets, including the regulation of embryonic development (officially, go_regulation_of_embryonic_development) gene set (refer to the caption of Fig 7 and to the Main Text for an explanation) (S11 Table). This gene set, which contained 133 genes (note that, 127 of these genes had a score with both models, and that the other six genes did not have a score with either model), required the removal of the 13 top-gaining genes from the gene set (namely and in order, PHLDA2, TRAF3IP1, LAMA5, HNF4A, BMP4, LFNG, FGFR1, TGIF2, WDPCP, CCSAP, TBX2, MBP, and STK4) before the gain for the gene set itself was lost. Though the biological relevance of this gene set is not be directly obvious, it may reflect the role of basic cellular processes (growth, differentiation and movement) in cancer [92]. (C) Remarkable gains from augmentation were observed for three of the genes mentioned (PHLDA2 via an eQTL-supported regulatory element ~750kb downstream from the transcription start-site; TRAF3IP1 via regulatory elements ~900kb upstream from the transcription start-site; LAMA5 via regulatory elements ~1400kb upstream from the transcription start-site) (refer [file pcbi.1009908.s008.pdf]

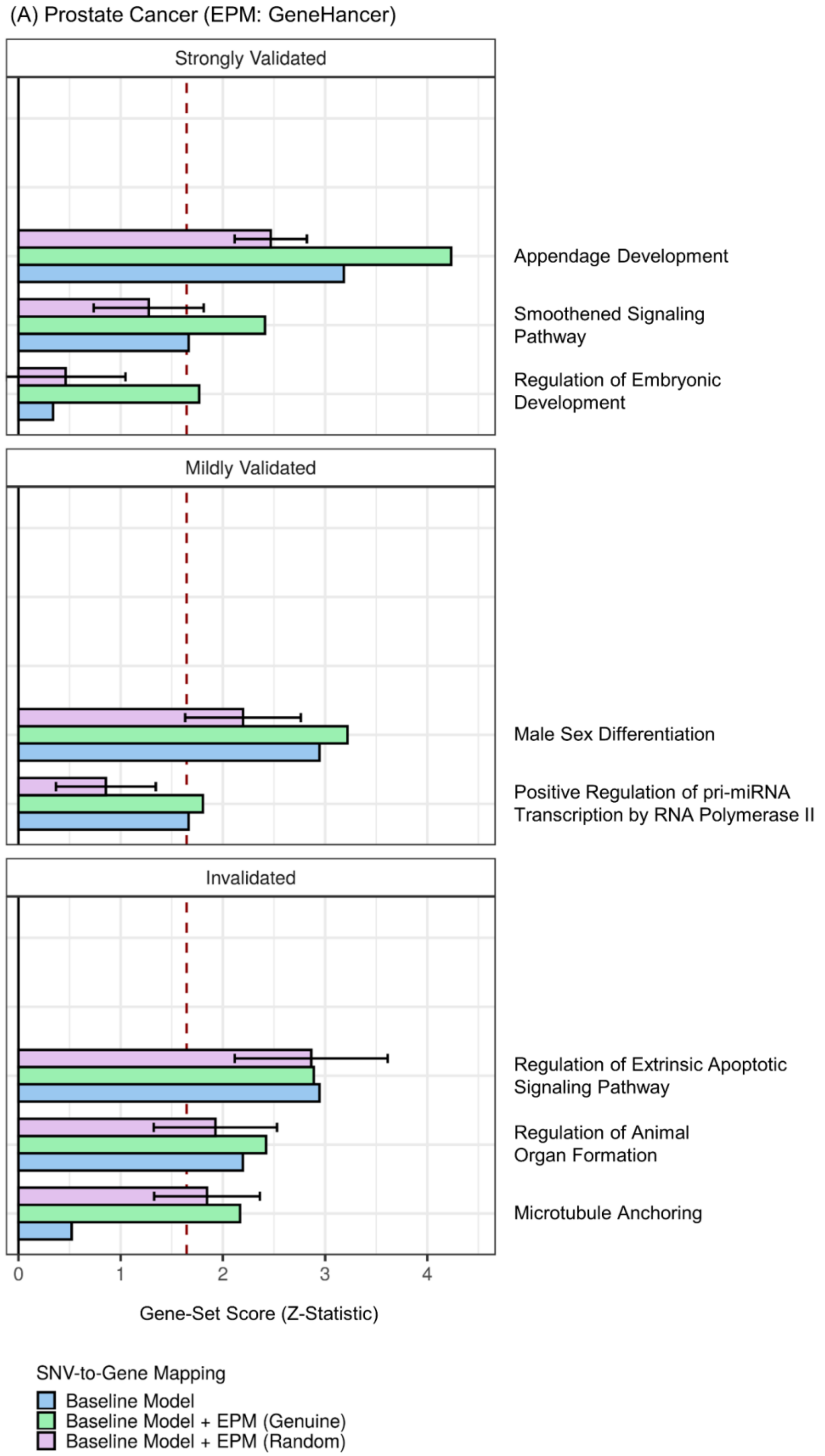
(A) Prostate Cancer (EPM: GeneHancer)
Strongly Validated
Appendage Development
Smoothened Signaling Pathway
Regulation of Embryonic Development
Mildly Validated
Male Sex Differentiation
Positive Regulation of pri-miRNA Transcription by RNA Polymerase II
Invalidated
Regulation of Extrinsic Apoptotic Signaling Pathway
Regulation of Animal Organ Formation
Microtubule Anchoring
0
1
2
3
4
Gene-Set Score (Z-Statistic)
SNV-to-Gene Mapping
Baseline Model
Baseline Model + EPM (Genuine)
Baseline Model + EPM (Random)

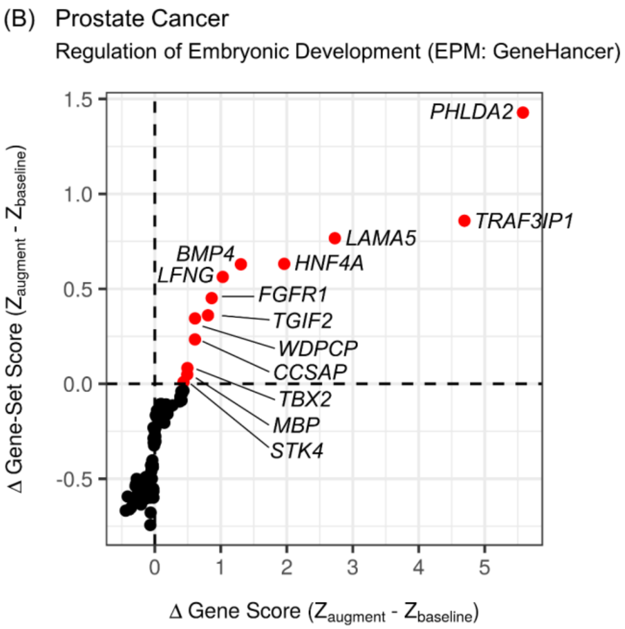
(B)
Prostate Cancer
Regulation of Embryonic Development (EPM: GeneHancer)
Δ Gene-Set Score ($Z_{augment}$ - $Z_{baseline}$)
Δ Gene Score ($Z_{augment}$ - $Z_{baseline}$)
1.5
1.0
0.5
0.0
-0.5
0
1
2
3
4
5
PHLDA2
TRAF3IP1
LAMA5
BMP4
HNF4A
LFNG
FGFR1
TGIF2
WDPCP
CCSAP
TBX2
MBP
STK4

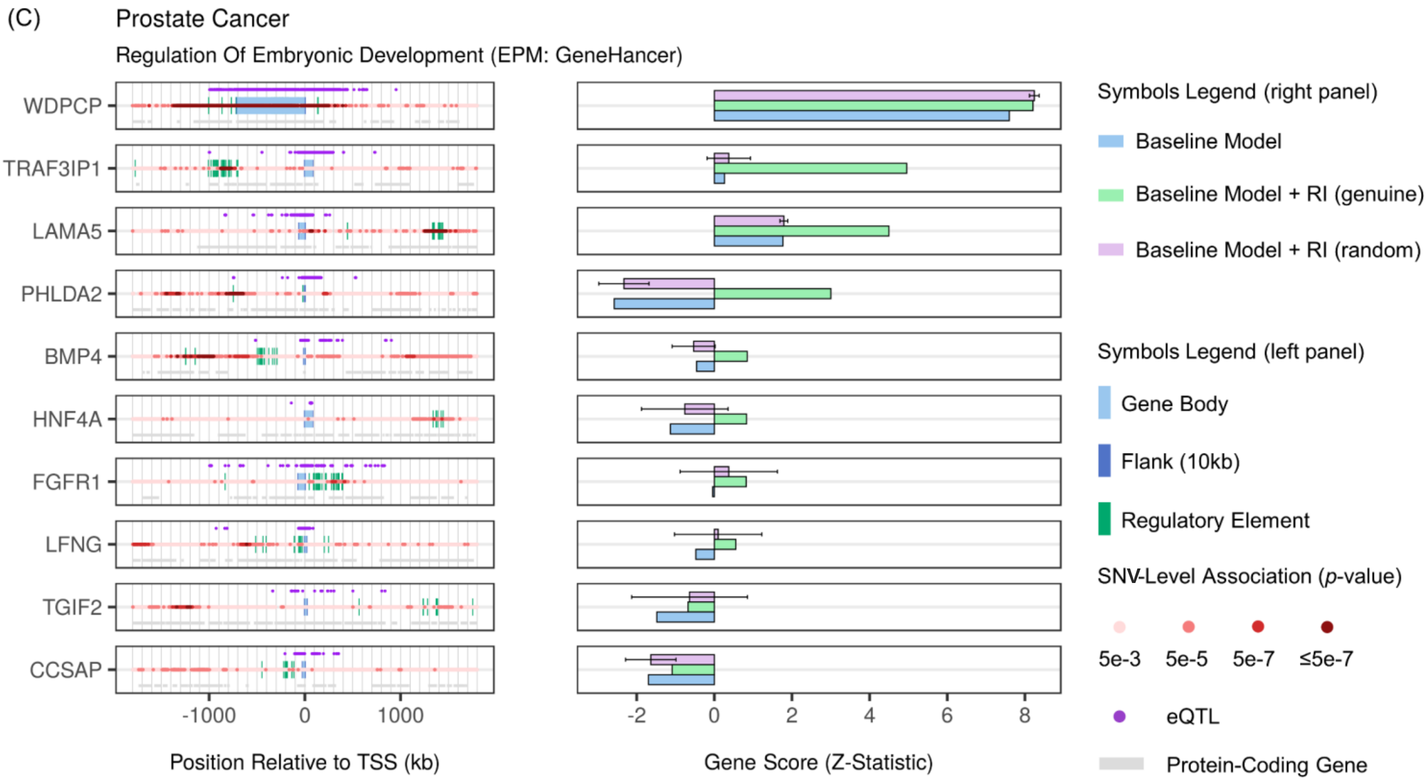
(C)
Prostate Cancer
Regulation Of Embryonic Development (EPM: GeneHancer)
WDPCP
TRAF3IP1
LAMA5
PHLDA2
BMP4
HNF4A
FGFR1
LFNG
TGIF2
CCSAP
-1000
0
1000
Position Relative to TSS (kb)
-2
0
2
4
6
8
Gene Score (Z-Statistic)
Symbols Legend (right panel)
Baseline Model
Baseline Model + RI (genuine)
Baseline Model + RI (random)
Symbols Legend (left panel)
Gene Body
Flank (10kb)
Regulatory Element
SNV-Level Association (*p*-value)
5e-3
5e-5
5e-7
≤5e-7
eQTL
Protein-Coding Gene
