# Supplementary material for: Incorporating regulatory interactions into gene-set analyses for GWAS data: A controlled analysis with the MAGMA tool
Source: PLoS Comput Biol. 2022 Mar 22;18(3):e1009908. doi: 10.1371/journal.pcbi.1009908 (PMC8939811; doi:10.1371/journal.pcbi.1009908)
Supplement: S1 Table — (DOCX) [file pcbi.1009908.s009.docx]

**Table A.** Overview of pc-HiC datasets selected for each phenotype.

| **Phenotype^*^** | **Selected pc-HiC Dataset** | **Number of Regulatory Interactions^+^** | **Number of Genes^+^** | **Mean Size (bp) of Regulatory Elements^+, #^** | **Remarks^!^** |
| --- | --- | --- | --- | --- | --- |
| Alzheimer’s Disease | Neural-Progenitor Cells [1] | 15,853 | 6,307 | 6,640 | NPC |
| Atrial Fibrillation | Heart [1] | 33,783 | 10,290 | 6,034 | ∪(LV, RV, RA3) |
| Bone Density | Mesendoderm [1] | 43,504 | 9,932 | 6,138 | ME |
| Breast Cancer | Ovary [1] | 7,962 | 4,619 | 6,354 | OV2 |
| C-Artery Disease | Blood Vessels [1] | 37,046 | 11,523 | 5,978 | AO |
| Crohn’s Disease | Spleen [1] | 72,188 | 12,235 | 6,304 | SX |
| Mac. Degeneration | Liver [1] | 28,561 | 9,186 | 6,408 | LI11 |
| Prostate Cancer | Adrenal Gland [1] | 22,886 | 8,985 | 5,992 | AD2 |
| Schizophrenia | Brain [1] | 146,466 | 15,113 | 5,786 | ∪(FC, HCmerge) |
| Type-2 Diabetes | Pancreas [1] | 23,107 | 9,924 | 6,074 | PA |

^*^ Phenotype abbreviations: C-Artery Disease (coronary-artery disease) and Mac. Degeneration (Macular Degeneration).

^+^ Numbers before annotation with SNVs (that is, including regulatory elements within - or linked to genes overlapping with - the MHC region).

^#^ Calculated across all regulatory interactions (that is, the same regulatory element may be considered multiple times if linked to multiple genes).

^!^ Remarks specify bio-samples used from the original resource (∪ means that we considered a union of bio-samples).

**Table B.** Overview of cMap datasets selected for each phenotype.

| **Phenotype^*^** | **Selected cMap Dataset** | **Number of Regulatory Interactions^+^** | **Number of Genes^+^** | **Mean Size (bp) of Regulatory Elements^+, #^** | **Remarks^!^** |
| --- | --- | --- | --- | --- | --- |
| Alzheimer’s Disease | Brain-Neuronal Cells [2] | 137,505 | 15,597 | 6,044 | ∪(Ex-Neu, DG-Neu) |
| Atrial Fibrillation | Heart-Muscle Cells [3] | 333,358 | 15,095 | 1,237 | - |
| Bone Density | Pancreatic-Islet Cells [4] | 53,833 | 5,301 | 763 | ∩(Expressed, Active-P.) |
| Breast Cancer | Prostate-Epithelial Cells [5] | 10,320 | 3,559 | 1,887 | - |
| C-Artery Disease | Heart-Muscle Cells [3] | 333,358 | 15,095 | 1,237 | - |
| Crohn’s Disease | Blood Cells [6] | 700,488 | 16,895 | 3,960 | ∪(All 18 cell-types) |
| Mac. Degeneration | Blood Cells [6] | 700,488 | 16,895 | 3,960 | ∪(All 18 cell-types) |
| Prostate Cancer | Prostate-Epithelial Cells [5] | 10,320 | 3,559 | 1,887 | - |
| Schizophrenia | Brain-Neuronal Cells [2] | 137,505 | 15,597 | 6,044 | ∪(Ex-Neu, DG-Neu) |
| Type-2 Diabetes | Pancreatic-Islet Cells [4] | 53,833 | 5,301 | 763 | ∩(Expressed, Active-P.) |

^*^ Phenotype abbreviations: C-Artery Disease (coronary-artery disease) and Mac. Degeneration (Macular Degeneration).

^+^ Numbers before annotation with SNVs (that is, including regulatory elements within - or linked to genes overlapping with - the MHC region).

^#^ Calculated across all regulatory interactions (that is, the same regulatory element may be considered multiple times if linked to multiple genes).

^!^ Conditions or bio-samples used from the original resource to generate the dataset (∪ means union of datasets, ∩ means intersect of conditions specified).

**References**

1. Jung I, Schmitt A, Diao Y, Lee AJ, Liu T, Yang D, et al. A compendium of promoter-centered long-range chromatin interactions in the human genome. Nat Genet. 2019;51: 1442–1449. doi:10.1038/s41588-019-0494-8

2. Song M, Yang X, Ren X, Maliskova L, Li B, Jones IR, et al. Mapping cis-regulatory chromatin contacts in neural cells links neuropsychiatric disorder risk variants to target genes. Nat Genet. 2019;51: 1252–1262. doi:10.1038/s41588-019-0472-1

3. Montefiori LE, Sobreira DR, Sakabe NJ, Aneas I, Joslin AC, Hansen GT, et al. A promoter interaction map for cardiovascular disease genetics. Elife. 2018;7. doi:10.7554/eLife.35788

4. Miguel-Escalada I, Bonàs-Guarch S, Cebola I, Ponsa-Cobas J, Mendieta-Esteban J, Atla G, et al. Human pancreatic islet three-dimensional chromatin architecture provides insights into the genetics of type 2 diabetes. Nat Genet. 2019;51: 1137–1148. doi:10.1038/s41588-019-0457-0

5. Rhie SK, Perez AA, Lay FD, Schreiner S, Shi J, Polin J, et al. A high-resolution 3D epigenomic map reveals insights into the creation of the prostate cancer transcriptome. Nat Commun. 2019;10. doi:10.1038/s41467-019-12079-8

6. Javierre BM, Burren OS, Wilder SP, Kreuzhuber R, Hill SM, Sewitz S, et al. Lineage-Specific Genome Architecture Links Enhancers and Non-coding Disease Variants to Target Gene Promoters. Cell. 2016;167: 1369-1384.e19. doi:10.1016/j.cell.2016.09.037
