# Supplementary material for: Incorporating regulatory interactions into gene-set analyses for GWAS data: A controlled analysis with the MAGMA tool
Source: PLoS Comput Biol. 2022 Mar 22;18(3):e1009908. doi: 10.1371/journal.pcbi.1009908 (PMC8939811; doi:10.1371/journal.pcbi.1009908)
Supplement: S2 Table — (DOCX) [file pcbi.1009908.s010.docx]

**Table A.** Number of significant genes by baseline model without and with augmentation (regulatory interactions).

|  | Baseline | Baseline with Augmentation from Regulatory Interactions | | | | | | | | | |
| --- | --- | --- | --- | --- | --- | --- | --- | --- | --- | --- | --- |
|  | - | EPM | | | | | HiC | | pc-HiC | | cMap |
| Phenotype^*^ | - | DHS07 | FOCS | Gene  Hancer | JEME | PsychENCODE | Fetal Brain | Adult Brain | Selected | Global | Selected |
| Alzheimer’s Disease | 162 | 161 | 168 | 201 | 177 | 165 | 214 | 194 | 171 | 255 | 261 |
| Atrial Fibrillation | 475 | 477 | 476 | 524 | 505 | 493 | 663 | 583 | 518 | 796 | 612 |
| Bone Density | 2,786 | 2,831 | 2,817 | 2,992 | 2,877 | 2,842 | 3,603 | 3,274 | 2,991 | 4,125 | 2,805 |
| Breast Cancer | 701 | 713 | 703 | 795 | 729 | 719 | 1,039 | 950 | 721 | 1,281 | 750 |
| C-Artery Disease | 290 | 292 | 288 | 310 | 300 | 301 | 451 | 386 | 325 | 606 | 363 |
| Crohn’s Disease | 553 | 575 | 567 | 590 | 601 | 569 | 775 | 725 | 711 | 1,018 | 1,072 |
| Mac. Degeneration | 167 | 167 | 169 | 179 | 176 | 171 | 277 | 226 | 198 | 383 | 430 |
| Prostate Cancer | 693 | 719 | 694 | 786 | 734 | 727 | 1,109 | 943 | 773 | 1,446 | 737 |
| Schizophrenia | 882 | 889 | 888 | 907 | 915 | 899 | 1,215 | 1,160 | 1,129 | 1,300 | 1,072 |
| Type-2 Diabetes | 954 | 966 | 959 | 1,005 | 1,003 | 974 | 1,313 | 1,227 | 1,054 | 1,606 | 975 |

^*^ Phenotype abbreviations: C-Artery Disease (coronary-artery disease) and Mac. Degeneration (Macular Degeneration).

**Table B.** Number of significant genes by baseline model without and with augmentation (larger flanks).

|  | Baseline | Baseline with Augmentation from Larger Flanks^^^ | | | | | | |
| --- | --- | --- | --- | --- | --- | --- | --- | --- |
| Phenotype^*^ | - | U20D20 | U35D35 | U50D50 | U100D100 | U250D250 | U500D500 | U1000D1000 |
| Alzheimer’s Disease | 162 | 182 | 206 | 223 | 272 | 402 | 581 | 792 |
| Atrial Fibrillation | 475 | 494 | 535 | 582 | 748 | 1,109 | 1,445 | 1,936 |
| Bone Density | 2,786 | 2,885 | 3,024 | 3,132 | 3,703 | 4,626 | 5,408 | 6,250 |
| Breast Cancer | 701 | 760 | 833 | 903 | 1,098 | 1,584 | 2,091 | 2,681 |
| C-Artery Disease | 290 | 304 | 310 | 337 | 471 | 752 | 1,129 | 1,748 |
| Crohn’s Disease | 553 | 599 | 642 | 685 | 874 | 1,271 | 1,711 | 2,452 |
| Mac. Degeneration | 167 | 193 | 226 | 272 | 359 | 559 | 949 | 1,220 |
| Prostate Cancer | 693 | 752 | 833 | 906 | 1,116 | 1,678 | 2,393 | 3,132 |
| Schizophrenia | 882 | 906 | 916 | 917 | 1,119 | 1,422 | 1,826 | 2,286 |
| Type-2 Diabetes | 954 | 1,024 | 1,066 | 1,146 | 1,430 | 1,964 | 2,511 | 3,272 |

^*^ Phenotype abbreviations: C-Artery Disease (coronary-artery disease) and Mac. Degeneration (Macular Degeneration).

^^^ Flanks are reported as UX (U; upstream from the transcription start-site) and DY (Y; downstream from the transcription end-site), where X and Y are flank size in kb.
