# Supplementary material for: Incorporating regulatory interactions into gene-set analyses for GWAS data: A controlled analysis with the MAGMA tool
Source: PLoS Comput Biol. 2022 Mar 22;18(3):e1009908. doi: 10.1371/journal.pcbi.1009908 (PMC8939811; doi:10.1371/journal.pcbi.1009908)
Supplement: S3 Table — (DOCX) [file pcbi.1009908.s011.docx]

**Table A.** No. of novel (N), known (K), and lost (L), significant genes resulting from augmentation (regulatory interactions).

|  | | Baseline with Augmentation from Regulatory Interactions | | | | | | | | | |
| --- | --- | --- | --- | --- | --- | --- | --- | --- | --- | --- | --- |
|  |  | EPM | | | | | HiC | | pc-HiC | | cMap |
| Phenotype^*^ | Gr.^+^ | DHS07 | FOCS | Gene  Hancer | JEME | PsychEN  CODE | Fetal Brain | Adult Brain | Selected | Global | Selected |
| Alzheimer’s Disease | N | 1 | 6^a^ | 41^b^ | 15^a^ | 3 | 77^b^ | 55^a^ | 11^a^ | 113^c^ | 111^c^ |
|  | K | 160 | 162 | 160 | 162 | 162 | 137 | 139 | 160 | 142 | 150 |
|  | L | 2 | 0 | 2 | 0 | 0 | 25 | 23 | 2 | 20 | 12 |
| Atrial Fibrillation | N | 7 | 3 | 76^b^ | 45^a^ | 20^a^ | 238^c^ | 147^c^ | 63^b^ | 376^c^ | 163^c^ |
|  | K | 470 | 473 | 448 | 460 | 473 | 425 | 436 | 455 | 420 | 449 |
|  | L | 5 | 2 | 27 | 15 | 2 | 50 | 39 | 20 | 55 | 26 |
| Bone Density | N | 61^b^ | 43^a^ | 325^c^ | 166^b^ | 74^b^ | 1,027^c^ | 639^c^ | 314^c^ | 1,566^c^ | 51^a^ |
|  | K | 2,770 | 2,774 | 2,667 | 2,711 | 2,768 | 2,576 | 2,635 | 2,677 | 2,559 | 2,754 |
|  | L | 16 | 12 | 119 | 75 | 18 | 210 | 151 | 109 | 227 | 32 |
| Breast Cancer | N | 15^a^ | 9 | 115^c^ | 57^a^ | 23^a^ | 410^c^ | 294^c^ | 23^a^ | 643^c^ | 49^b^ |
|  | K | 698 | 694 | 680 | 672 | 696 | 629 | 656 | 698 | 638 | 701 |
|  | L | 3 | 7 | 21 | 29 | 5 | 72 | 45 | 3 | 63 | 0 |
| C-Artery Disease | N | 5 | 3 | 29^a^ | 23 | 11^a^ | 185^c^ | 113^c^ | 45^b^ | 344^c^ | 89^b^ |
|  | K | 287 | 285 | 281 | 277 | 290 | 266 | 273 | 280 | 262 | 274 |
|  | L | 3 | 5 | 9 | 13 | 0 | 24 | 17 | 10 | 28 | 16 |
| Crohn’s Disease | N | 24^b^ | 17^a^ | 59^a^ | 57^b^ | 17^a^ | 267^c^ | 196^c^ | 179^c^ | 512^c^ | 583^c^ |
|  | K | 551 | 550 | 531 | 544 | 552 | 508 | 529 | 532 | 506 | 489 |
|  | L | 2 | 3 | 22 | 9 | 1 | 45 | 24 | 21 | 47 | 64 |
| Mac. Degeneration | N | 3 | 2 | 18^a^ | 11^a^ | 4 | 117^c^ | 63^b^ | 31^b^ | 224^c^ | 273^c^ |
|  | K | 164 | 167 | 161 | 165 | 167 | 160 | 163 | 167 | 159 | 157 |
|  | L | 3 | 0 | 6 | 2 | 0 | 7 | 4 | 0 | 8 | 10 |
| Prostate Cancer | N | 28^b^ | 10 | 110^c^ | 59^b^ | 36^b^ | 472^c^ | 293^c^ | 93^c^ | 799^c^ | 45^b^ |
|  | K | 691 | 684 | 676 | 675 | 691 | 637 | 650 | 680 | 647 | 692 |
|  | L | 2 | 9 | 17 | 18 | 2 | 56 | 43 | 13 | 46 | 1 |
| Schizophrenia | N | 17 | 14 | 81^a^ | 68^a^ | 26^a^ | 429^c^ | 333^c^ | 323^c^ | 534^c^ | 267^c^ |
|  | K | 872 | 874 | 826 | 847 | 873 | 786 | 827 | 806 | 766 | 805 |
|  | L | 10 | 8 | 56 | 35 | 9 | 96 | 55 | 76 | 116 | 77 |
| Type-2 Diabetes | N | 20^a^ | 17 | 121^a^ | 83^b^ | 30^a^ | 479^c^ | 358^c^ | 135^b^ | 771^c^ | 35^a^ |
|  | K | 946 | 942 | 884 | 920 | 944 | 834 | 869 | 919 | 835 | 940 |
|  | L | 8 | 12 | 70 | 34 | 10 | 120 | 85 | 35 | 119 | 14 |

^*^ Phenotype abbreviations: C-Artery Disease (coronary-artery disease) and Mac. Degeneration (Macular Degeneration).

^+^ Novel (N; gene significant with the augmented model only) / Known (K; gene significant with both models) / Lost (L; gene significant with the baseline model only)

Binomial test for more novel (N) genes than lost (L) genes (against the null that either outcome is equally likely or that losing is more likely). No letter (*p* ≥ 0.05); ^a^ (*p* ≥ 1e-05); ^b^ (*p* ≥ 1e-15); ^c^ (the rest). All *p*-values were adjusted for multiple testing (FDR) across all mappings (Table A and B) within each phenotype separately.

**Table B.** No. of novel (N), known (K), and lost (L), significant genes resulting from augmentation (larger flanks).

|  | | Baseline with Augmentation from Larger Flanks^^^ | | | | | | |
| --- | --- | --- | --- | --- | --- | --- | --- | --- |
| Phenotype^*^ | Gr.^+^ | U20D20 | U35D35 | U50D50 | U100D100 | U250D250 | U500D500 | U1000D1000 |
| Alzheimer’s Disease | N | 26^a^ | 57^b^ | 72^b^ | 127^c^ | 259^c^ | 444^c^ | 661^c^ |
|  | K | 156 | 149 | 151 | 145 | 143 | 137 | 131 |
|  | L | 6 | 13 | 11 | 17 | 19 | 25 | 31 |
| Atrial Fibrillation | N | 47^a^ | 102^b^ | 148^b^ | 320^c^ | 705^c^ | 1,077^c^ | 1,604^c^ |
|  | K | 447 | 433 | 434 | 428 | 404 | 368 | 332 |
|  | L | 28 | 42 | 41 | 47 | 71 | 107 | 143 |
| Bone Density | N | 230^b^ | 415^c^ | 566^c^ | 1,159^c^ | 2,156^c^ | 3,071^c^ | 4,032^c^ |
|  | K | 2,655 | 2,609 | 2,566 | 2,544 | 2,470 | 2,337 | 2,218 |
|  | L | 131 | 177 | 220 | 242 | 316 | 449 | 568 |
| Breast Cancer | N | 96^b^ | 177^c^ | 241^c^ | 466^c^ | 962^c^ | 1,489^c^ | 2,117^c^ |
|  | K | 664 | 656 | 662 | 632 | 622 | 602 | 564 |
|  | L | 37 | 45 | 39 | 69 | 79 | 99 | 137 |
| C-Artery Disease | N | 32^a^ | 54^a^ | 92^a^ | 223^c^ | 515^c^ | 903^c^ | 1,539^c^ |
|  | K | 272 | 256 | 245 | 248 | 237 | 226 | 209 |
|  | L | 18 | 34 | 45 | 42 | 53 | 64 | 81 |
| Crohn’s Disease | N | 73^b^ | 130^b^ | 182^c^ | 377^c^ | 792^c^ | 1,250^c^ | 2,012^c^ |
|  | K | 526 | 512 | 503 | 497 | 479 | 461 | 440 |
|  | L | 27 | 41 | 50 | 56 | 74 | 92 | 113 |
| Mac. Degeneration | N | 32^a^ | 72^b^ | 115^c^ | 205^c^ | 410^c^ | 806^c^ | 1,081^c^ |
|  | K | 161 | 154 | 157 | 154 | 149 | 143 | 139 |
|  | L | 6 | 13 | 10 | 13 | 18 | 24 | 28 |
| Prostate Cancer | N | 104^b^ | 191^c^ | 273^c^ | 502^c^ | 1,071^c^ | 1,808^c^ | 2,589^c^ |
|  | K | 648 | 642 | 633 | 614 | 607 | 585 | 543 |
|  | L | 45 | 51 | 60 | 79 | 86 | 108 | 150 |
| Schizophrenia | N | 82^a^ | 125^a^ | 160^a^ | 376^c^ | 720^c^ | 1,180^c^ | 1,681^c^ |
|  | K | 824 | 791 | 757 | 743 | 702 | 646 | 605 |
|  | L | 58 | 91 | 125 | 139 | 180 | 236 | 277 |
| Type-2 Diabetes | N | 118^b^ | 201^b^ | 287^c^ | 593^c^ | 1,185^c^ | 1,805^c^ | 2,603^c^ |
|  | K | 906 | 865 | 859 | 837 | 779 | 706 | 669 |
|  | L | 48 | 89 | 95 | 117 | 175 | 248 | 285 |

^*^ Phenotype abbreviations: C-Artery Disease (coronary-artery disease) and Mac. Degeneration (Macular Degeneration).

^+^ Novel (N; gene significant with the augmented model only) / Known (K; gene significant with both models) / Lost (L; gene significant with the baseline model only)

^^^ Flanks are reported as UX (U; upstream from the transcription start-site) and DY (Y; downstream from the transcription end-site), where X and Y are flank size in kb.

Binomial test for more novel (N) genes than lost (L) genes (against the null that either outcome is equally likely or that losing is more likely). No letter (*p* ≥ 0.05); ^a^ (*p* ≥ 1e-05); ^b^ (*p* ≥ 1e-15); ^c^ (the rest). All *p*-values were adjusted for multiple testing (FDR) across all mappings (Table A and B) within each phenotype separately.
