# Supplementary material for: Incorporating regulatory interactions into gene-set analyses for GWAS data: A controlled analysis with the MAGMA tool
Source: PLoS Comput Biol. 2022 Mar 22;18(3):e1009908. doi: 10.1371/journal.pcbi.1009908 (PMC8939811; doi:10.1371/journal.pcbi.1009908)
Supplement: S4 Table — (DOCX) [file pcbi.1009908.s012.docx]

**Table A.** Number of significant genes detected by baseline model with and without augmentation (larger flanks) based on MAGMA’s unadjusted gene-scores.

|  | Total^^^ | Baseline | Baseline with Augmentation from Larger Flanks^!^ | | | | | | |
| --- | --- | --- | --- | --- | --- | --- | --- | --- | --- |
| Phenotype^*^ | - | - | U20D20 | U35D35 | U50D50 | U100D100 | U250D250 | U500D500 | U1000D1000 |
| Alzheimer’s Disease | 18,415 | 209 | 234 | 269 | 295 | 363 | 555 | 687 | 994 |
| Atrial Fibrillation | 18,478 | 953 | 1,032 | 1,150 | 1,280 | 1,645 | 2,657 | 4,141 | 6,468 |
| Bone Density | 18,240 | 7,769 | 8,182 | 8,759 | 9,294 | 10,729 | 13,326 | 15,433 | 17,098 |
| Breast Cancer | 17,882 | 1,635 | 1,799 | 1,989 | 2,157 | 2,695 | 4,245 | 5,927 | 8,874 |
| C-Artery Disease | 18,199 | 381 | 394 | 445 | 519 | 671 | 977 | 1,586 | 2,407 |
| Crohn’s Disease | 18,239 | 1,179 | 1,232 | 1,378 | 1,576 | 2,013 | 3,094 | 4,817 | 7,329 |
| Mac. Degeneration | 18,162 | 194 | 242 | 270 | 310 | 404 | 638 | 1,055 | 1,521 |
| Prostate Cancer | 18,532 | 1,325 | 1,446 | 1,590 | 1,758 | 2,244 | 3,486 | 5,083 | 6,778 |
| Schizophrenia | 17,883 | 2,775 | 2,950 | 3,289 | 3,571 | 4,505 | 6,646 | 9,399 | 13,106 |
| Type-2 Diabetes | 18,316 | 3,072 | 3,302 | 3,626 | 3,970 | 5,034 | 7,445 | 10,391 | 13,496 |

^*^ Phenotype abbreviations: C-Artery Disease (coronary-artery disease) and Mac. Degeneration (Macular Degeneration).

^^^ For each phenotype, we focused our analyses exclusively on genes with a score for every SNV-to-gene mapping in both MAGMA and Pascal.

^!^ Flanks are reported as UX (U; upstream from the transcription start-site) and DY (Y; downstream from the transcription end-site), where X and Y are flank size in kb.

**Table B.** Number of significant genes detected by baseline model with and without augmentation (larger flanks) based on Pascal’s gene-scores.

|  | Total^^^ | Baseline | Baseline with Augmentation from Larger Flanks^!,+^ | | | | | | |
| --- | --- | --- | --- | --- | --- | --- | --- | --- | --- |
| Phenotype^*^ | - | - | U20D20 | U35D35 | U50D50 | U100D100 | U250D250 | U500D500 | U1000D1000 |
| Alzheimer’s Disease | 18,415 | 208 | 230 | 260 | 280 | 327 | 471 | 618 | NA |
| Atrial Fibrillation | 18,478 | 939 | 1,008 | 1,091 | 1,194 | 1,462 | 2,249 | 3,556 | NA |
| Bone Density | 18,240 | 7,710 | 8,101 | 8,655 | 9,169 | 10,489 | 13,016 | 15,168 | NA |
| Breast Cancer | 17,882 | 1,617 | 1,765 | 1,922 | 2,071 | 2,479 | 3,714 | 5,215 | NA |
| C-Artery Disease | 18,199 | 372 | 386 | 429 | 463 | 616 | 830 | 1,380 | NA |
| Crohn’s Disease | 18,239 | 1,138 | 1,207 | 1,313 | 1,484 | 1,829 | 2,658 | 4,074 | NA |
| Mac. Degeneration | 18,162 | 186 | 218 | 248 | 275 | 353 | 491 | 833 | NA |
| Prostate Cancer | 18,532 | 1,293 | 1,424 | 1,552 | 1,656 | 2,029 | 3,153 | 4,664 | NA |
| Schizophrenia | 17,883 | 2,700 | 2,884 | 3,152 | 3,410 | 4,200 | 6,265 | 8,833 | NA |
| Type-2 Diabetes | 18,316 | 3,021 | 3,254 | 3,547 | 3,808 | 4,686 | 6,995 | 9,814 | NA |

^*^ Phenotype abbreviations: C-Artery Disease (coronary-artery disease) and Mac. Degeneration (Macular Degeneration).

^^^ For each phenotype, we focused our analyses exclusively on genes with a score for every SNV-to-gene mapping in both MAGMA and Pascal.

^!^ Flanks are reported as UX (U; upstream from the transcription start-site) and DY (Y; downstream from the transcription end-site), where X and Y are flank size in kb.

^+^ Unfinished runs (due to exceptional run times and spontaneous crashing) were not included in the counts and labelled with NA.

**Table C.** No. of novel (N), known (K), and lost (L), significant genes resulting from augmentation (larger flanks) based on MAGMA’s unadjusted gene-scores.

|  | | Baseline with Augmentation from Larger Flanks^^^ | | | | | | |
| --- | --- | --- | --- | --- | --- | --- | --- | --- |
| Phenotype^*^ | Gr.^+^ | U20D20 | U35D35 | U50D50 | U100D100 | U250D250 | U500D500 | U1000D1000 |
| Alzheimer’s Disease | N | 34^a^ | 69^b^ | 95^c^ | 167^c^ | 368^c^ | 508^c^ | 826^c^ |
|  | K | 200 | 200 | 200 | 196 | 187 | 179 | 168 |
|  | L | 9 | 9 | 9 | 13 | 22 | 30 | 41 |
| Atrial Fibrillation | N | 130^b^ | 265^c^ | 398^c^ | 773^c^ | 1,806^c^ | 3,315^c^ | 5,609^c^ |
|  | K | 902 | 885 | 882 | 872 | 851 | 826 | 859 |
|  | L | 51 | 68 | 71 | 81 | 102 | 127 | 94 |
| Bone Density | N | 591^c^ | 1,219^c^ | 1,773^c^ | 3,210^c^ | 5,764^c^ | 7,793^c^ | 9,391^c^ |
|  | K | 7,591 | 7,540 | 7,521 | 7,519 | 7,562 | 7,640 | 7,707 |
|  | L | 178 | 229 | 248 | 250 | 207 | 129 | 62 |
| Breast Cancer | N | 228^c^ | 463^c^ | 627^c^ | 1,201^c^ | 2,764^c^ | 4,472^c^ | 7,390^c^ |
|  | K | 1,571 | 1,526 | 1,530 | 1,494 | 1,481 | 1,455 | 1,484 |
|  | L | 64 | 109 | 105 | 141 | 154 | 180 | 151 |
| C-Artery Disease | N | 40 | 98^b^ | 177^c^ | 335^c^ | 661^c^ | 1,284^c^ | 2,108^c^ |
|  | K | 354 | 347 | 342 | 336 | 316 | 302 | 299 |
|  | L | 27 | 34 | 39 | 45 | 65 | 79 | 82 |
| Crohn’s Disease | N | 127^a^ | 294^c^ | 494^c^ | 945^c^ | 2,041^c^ | 3,780^c^ | 6,276^c^ |
|  | K | 1,105 | 1,084 | 1,082 | 1,068 | 1,053 | 1,037 | 1,053 |
|  | L | 74 | 95 | 97 | 111 | 126 | 142 | 126 |
| Mac. Degeneration | N | 57^b^ | 90^b^ | 128^c^ | 223^c^ | 467^c^ | 894^c^ | 1,359^c^ |
|  | K | 185 | 180 | 182 | 181 | 171 | 161 | 162 |
|  | L | 9 | 14 | 12 | 13 | 23 | 33 | 32 |
| Prostate Cancer | N | 176^c^ | 348^c^ | 521^c^ | 1,018^c^ | 2,264^c^ | 3,873^c^ | 5,594^c^ |
|  | K | 1,270 | 1,242 | 1,237 | 1,226 | 1,222 | 1,210 | 1,184 |
|  | L | 55 | 83 | 88 | 99 | 103 | 115 | 141 |
| Schizophrenia | N | 286^c^ | 650^c^ | 954^c^ | 1,905^c^ | 4,057^c^ | 6,787^c^ | 10,431^c^ |
|  | K | 2,664 | 2,639 | 2,617 | 2,600 | 2,589 | 2,612 | 2,675 |
|  | L | 111 | 136 | 158 | 175 | 186 | 163 | 100 |
| Type-2 Diabetes | N | 343^c^ | 723^c^ | 1,079^c^ | 2,149^c^ | 4,555^c^ | 7,465^c^ | 10,541^c^ |
|  | K | 2,959 | 2,903 | 2,891 | 2,885 | 2,890 | 2,926 | 2,955 |
|  | L | 113 | 169 | 181 | 187 | 182 | 146 | 117 |

^*^ Phenotype abbreviations: C-Artery Disease (coronary-artery disease) and Mac. Degeneration (Macular Degeneration).

^+^ Novel (N; gene significant with the augmented model only) / Known (K; gene significant with both models) / Lost (L; gene significant with the baseline model only)

^^^ Flanks are reported as UX (U; upstream from the transcription start-site) and DY (Y; downstream from the transcription end-site), where X and Y are flank size in kb.

Binomial test for more novel (N) genes than lost (L) genes (against the null that either outcome is equally likely or that losing is more likely). No letter (*p* ≥ 0.05); ^a^ (*p* ≥ 1e-05); ^b^ (*p* ≥ 1e-15); ^c^ (the rest). All *p*-values were adjusted for multiple testing (FDR) across all mappings within each phenotype separately.

**Table D.** No. of novel (N), known (K), and lost (L), significant genes resulting from augmentation (larger flanks) based on Pascal’s gene-scores.

|  | | Baseline with Augmentation from Larger Flanks^^,!^ | | | | | | |
| --- | --- | --- | --- | --- | --- | --- | --- | --- |
| Phenotype^*^ | Gr.^+^ | U20D20 | U35D35 | U50D50 | U100D100 | U250D250 | U500D500 | U1000D1000 |
| Alzheimer’s Disease | N | 30^a^ | 62^b^ | 83^b^ | 143^c^ | 301^c^ | 451^c^ | NA |
|  | K | 200 | 198 | 197 | 184 | 170 | 167 | NA |
|  | L | 8 | 10 | 11 | 24 | 38 | 41 | NA |
| Atrial Fibrillation | N | 122^b^ | 233^c^ | 342^c^ | 640^c^ | 1,450^c^ | 2,772^c^ | NA |
|  | K | 886 | 858 | 852 | 882 | 799 | 784 | NA |
|  | L | 53 | 81 | 87 | 117 | 140 | 155 | NA |
| Bone Density | N | 578^c^ | 1,179^c^ | 1,721^c^ | 3,056^c^ | 5,544^c^ | 7,615^c^ | NA |
|  | K | 7,523 | 7,476 | 7,448 | 7,433 | 7,472 | 7,553 | NA |
|  | L | 187 | 234 | 262 | 277 | 238 | 157 | NA |
| Breast Cancer | N | 210^c^ | 427^c^ | 586^c^ | 1,040^c^ | 2,305^c^ | 3,832^c^ | NA |
|  | K | 1,555 | 1,495 | 1,485 | 1,439 | 1,409 | 1,383 | NA |
|  | L | 62 | 122 | 132 | 178 | 208 | 234 | NA |
| C-Artery Disease | N | 41 | 94^b^ | 141^b^ | 294^c^ | 530^c^ | 1,096^c^ | NA |
|  | K | 345 | 335 | 322 | 322 | 300 | 284 | NA |
|  | L | 27 | 37 | 50 | 50 | 72 | 88 | NA |
| Crohn’s Disease | N | 140^b^ | 271^c^ | 438^c^ | 816^c^ | 1,670^c^ | 3,110^c^ | NA |
|  | K | 1,067 | 1,042 | 1,046 | 1,013 | 988 | 964 | NA |
|  | L | 71 | 96 | 92 | 125 | 150 | 174 | NA |
| Mac. Degeneration | N | 41^b^ | 77^b^ | 106^c^ | 180^c^ | 335^c^ | 686^c^ | NA |
|  | K | 177 | 171 | 169 | 173 | 156 | 147 | NA |
|  | L | 9 | 15 | 17 | 13 | 30 | 39 | NA |
| Prostate Cancer | N | 176^c^ | 333^c^ | 458^c^ | 871^c^ | 1,988^c^ | 3,499^c^ | NA |
|  | K | 1,248 | 1,219 | 1,198 | 1,158 | 1,165 | 1,165 | NA |
|  | L | 45 | 74 | 95 | 135 | 128 | 128 | NA |
| Schizophrenia | N | 281^c^ | 592^c^ | 885^c^ | 1,712^c^ | 3,801^c^ | 6,340^c^ | NA |
|  | K | 2,603 | 2,560 | 2,525 | 2,488 | 2,464 | 2,493 | NA |
|  | L | 97 | 140 | 175 | 212 | 236 | 207 | NA |
| Type-2 Diabetes | N | 342^c^ | 698^c^ | 976^c^ | 1,893^c^ | 4,200^c^ | 6,972^c^ | NA |
|  | K | 2,912 | 2,849 | 2,832 | 2,793 | 2,795 | 2,842 | NA |
|  | L | 109 | 172 | 189 | 228 | 226 | 179 | NA |

^*^ Phenotype abbreviations: C-Artery Disease (coronary-artery disease) and Mac. Degeneration (Macular Degeneration).

^+^ Novel (N; gene significant with the augmented model only) / Known (K; gene significant with both models) / Lost (L; gene significant with the baseline model only)

^^^ Flanks are reported as UX (U; upstream from the transcription start-site) and DY (Y; downstream from the transcription end-site), where X and Y are flank size in kb.

^!^ Unfinished runs (due to exceptional run times and spontaneous crashing) were not included in the counts and labelled with NA.

Binomial test for more novel (N) genes than lost (L) genes (against the null that either outcome is equally likely or that losing is more likely). No letter (*p* ≥ 0.05); ^a^ (*p* ≥ 1e-05); ^b^ (*p* ≥ 1e-15); ^c^ (the rest). All *p*-values were adjusted for multiple testing (FDR) across all mappings (except U1000D1000) within each phenotype separately.
