# Supplementary material for: Incorporating regulatory interactions into gene-set analyses for GWAS data: A controlled analysis with the MAGMA tool
Source: PLoS Comput Biol. 2022 Mar 22;18(3):e1009908. doi: 10.1371/journal.pcbi.1009908 (PMC8939811; doi:10.1371/journal.pcbi.1009908)
Supplement: S5 Table — (DOCX) [file pcbi.1009908.s013.docx]

**Table A.** No. of significant genes by baseline model without and with augmentation including EPVP.

|  | Baseline | Baseline with Augmentation from Regulatory Interactions and Larger Flanks | | | | | | | | | | | | | |
| --- | --- | --- | --- | --- | --- | --- | --- | --- | --- | --- | --- | --- | --- | --- | --- |
|  | - | EPM | | | | | | pc-HiC | | | | cMap | | Flanks^^^ | |
|  | - | GeneHancer | | JEME | | PsychENCODE | | Selected | | Global | | Selected | | U100D100 | |
| Phenotype^*^ | - | R | P^+^ | R | P^+^ | R | P^+^ | R | P^+^ | R | P^+^ | R | P^+^ | R | P^+^ |
| Alzheimer’s Disease | 162 | 201^a^ | 157^b^  (±5) | 177^a^ | 155^b^  (±5) | 165^a^ | 161^b^  (±1) | 171^a^ | 151^b^  (±7) | 255^a^ | 205  (±34) | 261^a^ | 117^b^  (±28) | 272^a^ | 158  (±32) |
| Atrial Fibrillation | 475 | 524^a^ | 463^b^  (±10) | 505^a^ | 454^b^  (±9) | 493^a^ | 475  (±4) | 518^a^ | 465^b^  (±17) | 796^a^ | 576  (±59) | 612^a^ | 460  (±45) | 748^a^ | 508  (±62) |
| Bone Density | 2,786 | 2,992^a^ | 2,850  (±34) | 2,877^a^ | 2,737^b^  (±31) | 2,842^a^ | 2,788  (±16) | 2,991^a^ | 2,826  (±41) | 4,125^a^ | 3,188  (±79) | 2,805^a^ | 2,796  (±13) | 3,703^a^ | 2,759  (±128) |
| Breast Cancer | 701 | 795^a^ | 708  (±13) | 729^a^ | 659^b^  (±15) | 719^a^ | 701  (±13) | 721^a^ | 694^b^  (±8) | 1,281^a^ | 873  (±60) | 750^a^ | 705  (±7) | 1,098^a^ | 703  (±55) |
| C-Artery Disease | 290 | 310^a^ | 270^b^  (±7) | 300^a^ | 259^b^  (±8) | 301^a^ | 284^b^  (±5) | 325^a^ | 257^b^  (±13) | 606^a^ | 370  (±40) | 363^a^ | 281  (±27) | 471^a^ | 335  (±54) |
| Crohn’s Disease | 553 | 590^a^ | 540^b^  (±8) | 601^a^ | 515^b^  (±7) | 569^a^ | 548^b^  (±5) | 711^a^ | 535^b^  (±20) | 1,018^a^ | 666  (±56) | 1,072^a^ | 683  (±68) | 874^a^ | 501^b^  (±42) |
| Mac. Degeneration | 167 | 179^a^ | 171  (±8) | 176^a^ | 162^b^  (±7) | 171^a^ | 166  (±6) | 198^a^ | 165  (±8) | 383^a^ | 257  (±39) | 430^a^ | 284  (±41) | 359^a^ | 226  (±30) |
| Prostate Cancer | 693 | 786^a^ | 712  (±17) | 734^a^ | 664^b^  (±11) | 727^a^ | 688^b^  (±8) | 773^a^ | 657^b^  (±13) | 1,446^a^ | 933  (±56) | 737^a^ | 691  (±7) | 1,116^a^ | 795  (±67) |
| Schizophrenia | 882 | 907^a^ | 807^b^  (±15) | 915^a^ | 764^b^  (±10) | 899^a^ | 870^b^  (±5) | 1,129^a^ | 777^b^  (±32) | 1,300^a^ | 827^b^  (±58) | 1,072^a^ | 663^b^  (±39) | 1,119^a^ | 697^b^  (±61) |
| Type-2 Diabetes | 954 | 1,005^a^ | 904^b^  (±13) | 1,003^a^ | 866^b^  (±17) | 974^a^ | 944^b^  (±6) | 1,054^a^ | 886^b^  (±21) | 1,606^a^ | 1,065  (±58) | 975^a^ | 943^b^  (±7) | 1,430^a^ | 908^b^  (±92) |

^*^ Phenotype abbreviations: C-Artery Disease (coronary-artery disease) and Mac. Degeneration (Macular Degeneration).

^+^ Values represent mean (± one standard deviation) for 20 independent permutations of EPVP (P) for comparison to the results with genuine signal (R).

^^^ Flanks are reported as UX (U; upstream from the transcription start-site) and DY (Y; downstream from the transcription end-site), where X and Y are flank size in kb.

We tested (T-test) for more significant genes with genuine augmentation than with matched, random augmentation (^a^ on a count in an R column indicates *p* < 0.05), as well as for more significant genes with the baseline model than with random augmentation of the baseline model (^b^ on a count in a P column indicates *p* < 0.05). For each of these scenarios separately, we adjusted all *p*-values (within each phenotype) for multiple testing (FDR) across all mappings.
