# Supplementary material for: Incorporating regulatory interactions into gene-set analyses for GWAS data: A controlled analysis with the MAGMA tool
Source: PLoS Comput Biol. 2022 Mar 22;18(3):e1009908. doi: 10.1371/journal.pcbi.1009908 (PMC8939811; doi:10.1371/journal.pcbi.1009908)
Supplement: S6 Table — (DOCX) [file pcbi.1009908.s014.docx]

**Table A.** Number of significant gene sets by baseline model without and with augmentation (regulatory interactions).

|  | Baseline | Baseline with Augmentation from Regulatory Interactions | | | | | | | | | |
| --- | --- | --- | --- | --- | --- | --- | --- | --- | --- | --- | --- |
|  | - | EPM | | | | | HiC | | pc-HiC | | cMap |
| Phenotype^*^ | - | DHS07 | FOCS | Gene  Hancer | JEME | PsychEN  CODE | Fetal Brain | Adult Brain | Selected | Global | Selected |
| Alzheimer’s Disease | 4 | 6 | 5 | 4 | 4 | 7 | 2 | 3^b^ | 9 | 0^c^ | 4^c^ |
| Atrial Fibrillation | 19 | 20 | 20 | 21 | 21 | 21^c^ | 11 | 16^c^ | 19^a^ | 15 | 18 |
| Bone Density | 34 | 26^c^ | 30^c^ | 25^c^ | 30 | 27^c^ | 24^c^ | 23^c^ | 33 | 14^c^ | 30 |
| Breast Cancer | 4 | 4 | 2^b^ | 2 | 3 | 3^b^ | 0^c^ | 2^c^ | 2 | 0^c^ | 0^c^ |
| C-Artery Disease | 4 | 4^c^ | 4 | 4^c^ | 6 | 4 | 2^c^ | 2^c^ | 6^c^ | 0^c^ | 5^c^ |
| Crohn’s Disease | 22 | 23 | 23 | 25 | 23 | 25 | 11^c^ | 19^c^ | 19^b^ | 11^c^ | 5^c^ |
| Mac. Degeneration | 0 | 1 | 0 | 0 | 0 | 0^c^ | 0^c^ | 0^c^ | 4^c^ | 0 | 0^c^ |
| Prostate Cancer | 5 | 5^c^ | 4^c^ | 8 | 5^c^ | 4^c^ | 2^c^ | 3^c^ | 5^c^ | 1^c^ | 4^c^ |
| Schizophrenia | 3 | 1^c^ | 1^c^ | 0 | 3^c^ | 1^c^ | 1^c^ | 3^c^ | 4 | 1^c^ | 0^c^ |
| Type-2 Diabetes | 7 | 6 | 4 | 11 | 7 | 7^b^ | 3^c^ | 3^c^ | 1^c^ | 1^c^ | 9 |

^*^ Phenotype abbreviations: C-Artery Disease (coronary-artery disease) and Mac. Degeneration (Macular Degeneration).

One-sided, paired Wilcoxon test for attenuation of gene-set scores with augmentation relative to the baseline. No letter (*p* ≥ 0.05); ^a^ (*p* ≥ 1e-05); ^b^ (*p* ≥ 1e-15); ^c^ (the rest). All *p*-values were adjusted for multiple testing (FDR) across all mappings (Table A and B) within each phenotype separately.

**Table B.** Number of significant gene sets by baseline model without and with augmentation (larger flanks).

|  | Baseline | Baseline with Augmentation from Larger Flanks^^^ | | | | | | |
| --- | --- | --- | --- | --- | --- | --- | --- | --- |
| Phenotype | - | U20D20 | U35D35 | U50D50 | U100D100 | U250D250 | U500D500 | U1000D1000 |
| Alzheimer’s Disease | 4 | 7 | 4^c^ | 0^c^ | 2 | 0 | 0 | 0 |
| Atrial Fibrillation | 19 | 18 | 19 | 16 | 12^b^ | 4^c^ | 0^c^ | 0^c^ |
| Bone Density | 34 | 27 | 30^a^ | 26^c^ | 20^c^ | 13^c^ | 1^c^ | 1^c^ |
| Breast Cancer | 4 | 0 | 0^c^ | 2^c^ | 2^c^ | 1^c^ | 0^c^ | 0^c^ |
| C-Artery Disease | 4 | 4^c^ | 3^c^ | 5^c^ | 0^c^ | 0^c^ | 0^c^ | 0^c^ |
| Crohn’s Disease | 22 | 17^c^ | 13^c^ | 15^c^ | 5^c^ | 0^c^ | 0^c^ | 0^c^ |
| Mac. Degeneration | 0 | 2 | 0 | 0^c^ | 0^c^ | 0^c^ | 0^c^ | 0^c^ |
| Prostate Cancer | 5 | 3^c^ | 3^c^ | 6^c^ | 0^c^ | 0^c^ | 0^c^ | 0^c^ |
| Schizophrenia | 3 | 1^c^ | 0^c^ | 1 | 0^c^ | 0^c^ | 0 | 0^c^ |
| Type-2 Diabetes | 7 | 9 | 4 | 2^c^ | 0^c^ | 0^c^ | 0^c^ | 0^c^ |

^*^ Phenotype abbreviations: C-Artery Disease (coronary-artery disease) and Mac. Degeneration (Macular Degeneration).

^^^ Flanks are reported as UX (U; upstream from the transcription start-site) and DY (Y; downstream from the transcription end-site), where X and Y are flank size in kb.

One-sided, paired Wilcoxon test for attenuation of gene-set scores with augmentation relative to the baseline. No letter (*p* ≥ 0.05); ^a^ (*p* ≥ 1e-05); ^b^ (*p* ≥ 1e-15); ^c^ (the rest). All *p*-values were adjusted for multiple testing (FDR) across all mappings (Table A and B) within each phenotype separately.
