# Supplementary material for: Incorporating regulatory interactions into gene-set analyses for GWAS data: A controlled analysis with the MAGMA tool
Source: PLoS Comput Biol. 2022 Mar 22;18(3):e1009908. doi: 10.1371/journal.pcbi.1009908 (PMC8939811; doi:10.1371/journal.pcbi.1009908)
Supplement: S7 Table — (DOCX) [file pcbi.1009908.s015.docx]

**Table A.** No. of novel (N), known (K), and lost (L), significant gene sets resulting from augmentation (regulatory interactions).

|  | | Baseline with Augmentation from Regulatory Interactions^!^ | | | | | | | | | |
| --- | --- | --- | --- | --- | --- | --- | --- | --- | --- | --- | --- |
|  |  | EPM | | | | | HiC | | pc-HiC | | cMap |
| Phenotype^*^ | Gr.^+^ | DHS07 | FOCS | Gene  Hancer | JEME | PsychEN  CODE | Fetal Brain | Adult Brain | Selected | Global | Selected |
| Alzheimer’s Disease | N | 2 | 2 | 1 | 1 | 3 | 0 | 0 | 5 | 0 | 1 |
|  | K | 4 | 3 | 3 | 3 | 4 | 4 | 3 | 4 | 0 | 3 |
|  | L | 0 | 1 | 1 | 1 | 0 | 2 | 1 | 0 | 4 | 1 |
| Atrial Fibrillation | N | 2 | 1 | 5 | 3 | 3 | 2 | 3 | 3 | 3 | 2 |
|  | K | 21 | 21 | 19 | 25 | 20 | 14 | 19 | 21 | 18 | 21 |
|  | L | 1 | 0 | 4 | 1 | 0 | 9 | 6 | 4 | 6 | 4 |
| Bone Density | N | 0 | 0 | 4 | 4 | 0 | 4 | 4 | 3 | 2 | 0 |
|  | K | 32 | 38 | 30 | 32 | 34 | 25 | 27 | 38 | 17 | 34 |
|  | L | 6 | 4 | 11 | 9 | 7 | 16 | 14 | 6 | 23 | 5 |
| Breast Cancer | N | 0 | 0 | 2 | 2 | 0 | 0 | 1 | 1 | 0 | 0 |
|  | K | 5 | 2 | 0 | 1 | 4 | 0 | 1 | 1 | 0 | 0 |
|  | L | 0 | 3 | 4 | 3 | 1 | 4 | 3 | 3 | 4 | 4 |
| C-Artery Disease | N | 0 | 0 | 2 | 2 | 0 | 0 | 1 | 2 | 0 | 2 |
|  | K | 4 | 4 | 3 | 4 | 4 | 3 | 2 | 4 | 0 | 3 |
|  | L | 0 | 0 | 2 | 0 | 0 | 2 | 3 | 0 | 4 | 1 |
| Crohn’s Disease | N | 1 | 2 | 9 | 2 | 6 | 2 | 3 | 2 | 5 | 2 |
|  | K | 27 | 29 | 22 | 26 | 24 | 12 | 20 | 24 | 9 | 3 |
|  | L | 2 | 2 | 8 | 4 | 2 | 14 | 8 | 6 | 17 | 21 |
| Mac. Degeneration | N | 1 | 0 | 0 | 0 | 0 | 0 | 0 | 4 | 0 | 0 |
|  | K | 0 | 0 | 0 | 0 | 0 | 0 | 0 | 0 | 0 | 0 |
|  | L | 0 | 0 | 0 | 0 | 0 | 0 | 0 | 0 | 0 | 0 |
| Prostate Cancer | N | 1 | 0 | 2 | 1 | 0 | 0 | 0 | 2 | 1 | 0 |
|  | K | 5 | 4 | 8 | 6 | 4 | 2 | 3 | 4 | 0 | 4 |
|  | L | 1 | 1 | 0 | 1 | 1 | 5 | 4 | 2 | 5 | 1 |
| Schizophrenia | N | 0 | 0 | 0 | 2 | 0 | 0 | 2 | 3 | 0 | 0 |
|  | K | 1 | 1 | 0 | 1 | 1 | 1 | 1 | 1 | 1 | 0 |
|  | L | 2 | 2 | 3 | 2 | 2 | 2 | 2 | 2 | 2 | 3 |
| Type-2 Diabetes | N | 0 | 0 | 4 | 2 | 0 | 1 | 1 | 0 | 0 | 4 |
|  | K | 7 | 4 | 7 | 7 | 9 | 2 | 2 | 1 | 1 | 8 |
|  | L | 2 | 3 | 0 | 2 | 0 | 5 | 5 | 6 | 6 | 0 |

^*^ Phenotype abbreviations: C-Artery Disease (coronary-artery disease) and Mac. Degeneration (Macular Degeneration).

^+^ Novel (N; a gene set significant with the augmented model only)

Known (K; a gene set significant with both models)

Lost (L; a gene set significant with the baseline model only)

^!^ Counts do not simply relate back to simple gene-set counts (Table A in S6 Table) due to requirement to merge some “rrvgo” results for this analysis (see Methods).

**Table B.** No. of novel (N), known (K), and lost (L), significant gene sets resulting from augmentation (larger flanks).

|  | | Baseline with Augmentation from Larger Flanks^^,!^ | | | | | | |
| --- | --- | --- | --- | --- | --- | --- | --- | --- |
| Phenotype^*^ | Gr.^+^ | U20D20 | U35D35 | U50D50 | U100D100 | U250D250 | U500D500 | U1000D1000 |
| Alzheimer’s Disease | N | 3 | 1 | 0 | 1 | 0 | 0 | 0 |
|  | K | 4 | 3 | 0 | 3 | 0 | 0 | 0 |
|  | L | 0 | 1 | 4 | 2 | 4 | 4 | 4 |
| Atrial Fibrillation | N | 1 | 4 | 3 | 3 | 0 | 0 | 0 |
|  | K | 23 | 20 | 18 | 15 | 5 | 0 | 0 |
|  | L | 2 | 3 | 7 | 10 | 16 | 19 | 19 |
| Bone Density | N | 2 | 6 | 4 | 5 | 1 | 0 | 0 |
|  | K | 34 | 32 | 28 | 21 | 16 | 1 | 1 |
|  | L | 11 | 12 | 15 | 18 | 25 | 34 | 33 |
| Breast Cancer | N | 0 | 0 | 1 | 2 | 1 | 0 | 0 |
|  | K | 0 | 0 | 1 | 0 | 0 | 0 | 0 |
|  | L | 4 | 4 | 4 | 4 | 4 | 4 | 4 |
| C-Artery Disease | N | 1 | 1 | 3 | 0 | 0 | 0 | 0 |
|  | K | 3 | 3 | 2 | 0 | 0 | 0 | 0 |
|  | L | 1 | 2 | 2 | 4 | 4 | 4 | 4 |
| Crohn’s Disease | N | 5 | 3 | 2 | 1 | 0 | 0 | 0 |
|  | K | 17 | 13 | 18 | 7 | 0 | 0 | 0 |
|  | L | 11 | 13 | 11 | 18 | 22 | 22 | 22 |
| Mac. Degeneration | N | 2 | 0 | 0 | 0 | 0 | 0 | 0 |
|  | K | 0 | 0 | 0 | 0 | 0 | 0 | 0 |
|  | L | 0 | 0 | 0 | 0 | 0 | 0 | 0 |
| Prostate Cancer | N | 0 | 0 | 3 | 0 | 0 | 0 | 0 |
|  | K | 4 | 3 | 5 | 0 | 0 | 0 | 0 |
|  | L | 3 | 3 | 2 | 5 | 5 | 5 | 5 |
| Schizophrenia | N | 0 | 0 | 0 | 0 | 0 | 0 | 0 |
|  | K | 1 | 0 | 1 | 0 | 0 | 0 | 0 |
|  | L | 2 | 3 | 2 | 3 | 3 | 3 | 3 |
| Type-2 Diabetes | N | 5 | 2 | 1 | 0 | 0 | 0 | 0 |
|  | K | 5 | 2 | 1 | 0 | 0 | 0 | 0 |
|  | L | 2 | 5 | 6 | 7 | 7 | 7 | 7 |

^*^ Phenotype abbreviations: C-Artery Disease (coronary-artery disease) and Mac. Degeneration (Macular Degeneration).

^+^ Novel (a gene set significant with the augmented model only)

Known (a gene set significant with both models)

Lost (a gene set significant with the baseline model only)

^^^ Flanks are reported as UX (U; upstream from the transcription start-site) and DY (Y; downstream from the transcription end-site), where X and Y are flank size in kb.

^!^ Counts do not simply relate back to simple gene-set counts (Table B in S6 Table) due to requirement to merge some “rrvgo” results for this analysis (see Methods).
