# Supplementary material for: Incorporating regulatory interactions into gene-set analyses for GWAS data: A controlled analysis with the MAGMA tool
Source: PLoS Comput Biol. 2022 Mar 22;18(3):e1009908. doi: 10.1371/journal.pcbi.1009908 (PMC8939811; doi:10.1371/journal.pcbi.1009908)
Supplement: S8 Table — (DOCX) [file pcbi.1009908.s016.docx]

**Table A.** No. of gaining and non-gaining gene sets amongst significant gene sets detected using baseline model with augmentation.

|  | | Baseline with Augmentation from Regulatory Interactions and Large Flanks | | | | | | |
| --- | --- | --- | --- | --- | --- | --- | --- | --- |
|  |  | EPM | | | pc-HiC | | cMap | Flanks^^^ |
| Phenotype^*^ | >/≤ | GeneHancer | JEME | PsychENCODE | Selected | Global | Selected | U100D100 |
| Alzheimer’s Disease | > | 2 | 1 | 4 | 9 | 0 | 1 | 1 |
|  | ≤ | 2 | 3 | 3 | 0 | 0 | 3 | 1 |
| Atrial Fibrillation | > | 10 | 14 | 9 | 10 | 6 | 8 | 4 |
|  | ≤ | 11 | 7 | 12 | 9 | 9 | 10 | 8 |
| Bone Density | > | 6 | 23 | 7 | 25 | 4 | 8 | 10 |
|  | ≤ | 19 | 7 | 20 | 8 | 10 | 22 | 10 |
| Breast Cancer | > | 2 | 3 | 2 | 1 | 0 | 0 | 2 |
|  | ≤ | 0 | 0 | 1 | 1 | 0 | 0 | 0 |
| C-Artery Disease | > | 2 | 4 | 1 | 2 | 0 | 2 | 0 |
|  | ≤ | 2 | 2 | 3 | 4 | 0 | 3 | 0 |
| Crohn’s Disease | > | 17 | 13 | 20 | 5 | 5 | 3 | 3 |
|  | ≤ | 8 | 10 | 5 | 14 | 6 | 2 | 2 |
| Mac. Degeneration | > | 0 | 0 | 0 | 4 | 0 | 0 | 0 |
|  | ≤ | 0 | 0 | 0 | 0 | 0 | 0 | 0 |
| Prostate Cancer | > | 7 | 5 | 1 | 3 | 1 | 0 | 0 |
|  | ≤ | 1 | 0 | 3 | 2 | 0 | 4 | 0 |
| Schizophrenia | > | 0 | 3 | 0 | 4 | 1 | 0 | 0 |
|  | ≤ | 0 | 0 | 1 | 0 | 0 | 0 | 0 |
| Type-2 Diabetes | > | 10 | 7 | 3 | 0 | 1 | 9 | 0 |
|  | ≤ | 1 | 0 | 4 | 1 | 0 | 0 | 0 |

^*^ Phenotype abbreviations: C-Artery Disease (coronary-artery disease) and Mac. Degeneration (Macular Degeneration).

>/≤ divides gene sets performing better (>) with augmentation of the baseline model compared to baseline model itself (a gain), from the rest (≤).

^^^ Flanks are reported as UX (U; upstream from the transcription start-site) and DY (Y; downstream from the transcription end-site), where X and Y are flank size in kb.
