# Supplementary material for: Incorporating regulatory interactions into gene-set analyses for GWAS data: A controlled analysis with the MAGMA tool
Source: PLoS Comput Biol. 2022 Mar 22;18(3):e1009908. doi: 10.1371/journal.pcbi.1009908 (PMC8939811; doi:10.1371/journal.pcbi.1009908)
Supplement: S9 Table — (DOCX) [file pcbi.1009908.s017.docx]

**Table A.** No. of strongly validated, mildly validated and invalidated gains amongst significant gene sets that gained from augmentation of the baseline model.

|  | Baseline with Augmentation from Regulatory Interactions and Large Flanks | | | | | | | | | | | | | | | | | | | | |
| --- | --- | --- | --- | --- | --- | --- | --- | --- | --- | --- | --- | --- | --- | --- | --- | --- | --- | --- | --- | --- | --- |
|  | EPM | | | | | | | | | pc-HiC | | | | | | cMap | | | Flanks^^^ | | |
|  | GeneHancer | | | JEME | | | PsychENCODE | | | Selected | | | Global | | | Selected | | | U100D100 | | |
| Phenotype^*^ | ++ | + | - | ++ | + | - | ++ | + | - | ++ | + | - | ++ | + | - | ++ | + | - | ++ | + | - |
| Alzheimer’s Disease | 0 | 1 | 1 | 0 | 1 | 0 | 0 | 0 | 4 | 1 | 5 | 3 | 0 | 0 | 0 | 1 | 0 | 0 | 1 | 0 | 0 |
| Atrial Fibrillation | 2 | 2 | 6 | 4 | 6 | 4 | 3 | 1 | 5 | 2 | 3 | 5 | 1 | 2 | 3 | 0 | 1 | 7 | 1 | 2 | 1 |
| Bone Density | 0 | 3 | 3 | 7 | 6 | 10 | 2 | 2 | 3 | 7 | 6 | 12 | 3 | 1 | 0 | 0 | 3 | 5 | 8 | 2 | 0 |
| Breast Cancer | 1 | 0 | 1 | 3 | 0 | 0 | 0 | 1 | 1 | 1 | 0 | 0 | 0 | 0 | 0 | 0 | 0 | 0 | 2 | 0 | 0 |
| C-Artery Disease | 1 | 0 | 1 | 3 | 1 | 0 | 0 | 0 | 1 | 0 | 1 | 1 | 0 | 0 | 0 | 1 | 1 | 0 | 0 | 0 | 0 |
| Crohn’s Disease | 10 | 5 | 2 | 2 | 4 | 7 | 6 | 6 | 8 | 3 | 0 | 2 | 4 | 0 | 1 | 1 | 0 | 2 | 2 | 1 | 0 |
| Mac. Degeneration | 0 | 0 | 0 | 0 | 0 | 0 | 0 | 0 | 0 | 4 | 0 | 0 | 0 | 0 | 0 | 0 | 0 | 0 | 0 | 0 | 0 |
| Prostate Cancer | 3 | 2 | 2 | 1 | 1 | 3 | 0 | 0 | 1 | 1 | 1 | 1 | 1 | 0 | 0 | 0 | 0 | 0 | 0 | 0 | 0 |
| Schizophrenia | 0 | 0 | 0 | 1 | 1 | 1 | 0 | 0 | 0 | 2 | 2 | 0 | 0 | 0 | 1 | 0 | 0 | 0 | 0 | 0 | 0 |
| Type-2 Diabetes | 2 | 7 | 1 | 2 | 2 | 3 | 0 | 0 | 3 | 0 | 0 | 0 | 1 | 0 | 0 | 1 | 4 | 4 | 0 | 0 | 0 |

^*^ Phenotype abbreviations: C-Artery Disease (coronary-artery disease) and Mac. Degeneration (Macular Degeneration).

++ (strongly validated), + (mildly validated), - (invalidated)

^^^ Flanks are reported as UX (U; upstream from the transcription start-site) and DY (Y; downstream from the transcription end-site), where X and Y are flank size in kb.
