# Supplementary material for: Incorporating regulatory interactions into gene-set analyses for GWAS data: A controlled analysis with the MAGMA tool
Source: PLoS Comput Biol. 2022 Mar 22;18(3):e1009908. doi: 10.1371/journal.pcbi.1009908 (PMC8939811; doi:10.1371/journal.pcbi.1009908)
Supplement: S10 Table — (DOCX) [file pcbi.1009908.s018.docx]

**Table A.** No. of robust and non-robust gains amongst significant gene sets with validated (mildly and strongly) gains from augmentation of the baseline model.

|  | | | Baseline with Augmentation from Regulatory Interactions and Large Flanks | | | | | | |
| --- | --- | --- | --- | --- | --- | --- | --- | --- | --- |
|  |  |  | EPM | | | pc-HiC | | cMap | Flanks^^^ |
| Phenotype^*^ | IR^!^ | | Gene  Hancer | JEME | PsychEN  CODE | Selected | Global | Selected | U100D100 |
| Alzheimer’s Disease | R | 1 | | 1 | 0 | 5 | 0 | 1 | 1 |
|  | NR | 0 | | 0 | 0 | 1 | 0 | 0 | 0 |
| Atrial Fibrillation | R | 3 | | 9 | 2 | 1 | 3 | 1 | 2 |
|  | NR | 1 | | 1 | 2 | 4 | 0 | 0 | 1 |
| Bone Density | R | 3 | | 9 | 1 | 7 | 1 | 1 | 7 |
|  | NR | 0 | | 4 | 3 | 6 | 3 | 2 | 3 |
| Breast Cancer | R | 1 | | 1 | 0 | 0 | 0 | 0 | 0 |
|  | NR | 0 | | 2 | 1 | 1 | 0 | 0 | 2 |
| C-Artery Disease | R | 0 | | 3 | 0 | 1 | 0 | 1 | 0 |
|  | NR | 1 | | 1 | 0 | 0 | 0 | 1 | 0 |
| Crohn’s Disease | R | 6 | | 5 | 10 | 1 | 4 | 0 | 0 |
|  | NR | 9 | | 1 | 2 | 2 | 0 | 1 | 3 |
| Mac. Degeneration | R | 0 | | 0 | 0 | 1 | 0 | 0 | 0 |
|  | NR | 0 | | 0 | 0 | 3 | 0 | 0 | 0 |
| Prostate Cancer | R | 3 | | 1 | 0 | 0 | 0 | 0 | 0 |
|  | NR | 2 | | 1 | 0 | 2 | 1 | 0 | 0 |
| Schizophrenia | R | 0 | | 0 | 0 | 2 | 0 | 0 | 0 |
|  | NR | 0 | | 2 | 0 | 2 | 0 | 0 | 0 |
| Type-2 Diabetes | R | 6 | | 2 | 0 | 0 | 0 | 3 | 0 |
|  | NR | 2 | | 1 | 0 | 0 | 1 | 2 | 0 |

^*^ Phenotype abbreviations: C-Artery Disease (coronary-artery disease) and Mac. Degeneration (Macular Degeneration).

^!^ IR (iterative reduction) column divides gene sets based on if they demonstrated a robust (R) or non-robust (NR) gain.

^^^ Flanks are reported as UX (U; upstream from the transcription start-site) and DY (Y; downstream from the transcription end-site), where X and Y are flank size in kb.
