# Supplementary material for: Incorporating regulatory interactions into gene-set analyses for GWAS data: A controlled analysis with the MAGMA tool
Source: PLoS Comput Biol. 2022 Mar 22;18(3):e1009908. doi: 10.1371/journal.pcbi.1009908 (PMC8939811; doi:10.1371/journal.pcbi.1009908)
Supplement: Attachment — Submitted filename: response_letter.docx [file pcbi.1009908.s020.docx]

Dear Editors,

First, we would like to thank you and the referees for your time and effort in reviewing our manuscript. We appreciate the comments raised by the referees. They were very constructive and helped us improve our manuscript. We are submitting a revised manuscript that addresses all the issues raised by the referees. New sections added to the manuscript and other changes are marked in blue.

Please find below our point-by-point response to the referees' comments.

Yours sincerely,

Prof. Rani Elkon

Department of Human Molecular Genetics & Biochemistry

Sackler School of Medicine

Tel Aviv University

Tel Aviv 69978, Israel

-------------------------------------------------------------------------------------------------------------------------------

Reviewer 1

-------------------------------------------------------------------------------------------------------------------------------

**Reviewer #1: The manuscripts seeks to systematically evaluate the effect of different types of augmentation (based on flanks and based on regulatory interactions / RIs) on SNV-to-gene mapping and gene set characterization performed for GWAS study. The authors consider multiple GWAS studies and multiple RI datasets to assess this. The design of the study overall is well done and the study is interesting and insightful. I have some comments that I hope can strengthen the study.**

We thank the referee for the positive feedback and the valuable comments.

**1- The main goal of the paper is to assess the effect of augmenting the GWAS gene set enrichment analysis with regulatory interactions. In the introduction, the authors talk about different methods that achieve this goal using different strategies. However, as they explain the results, it is unclear what augmentation method they use. For example, the second subsection of Results discusses the effect of augmentation on number of significant genes identified, yet I could not find what method was used to incorporate RIs to augment. While "Methods" describes some details, it is still important to conceptually describe how RIs are used to augment the analysis. Is this augmentation similar to H-MAGMA, similar to e-MAGMA or some other approach? If those above, still more information is needed to describe how this RI augmentation is performed and what it corresponds to.**

Augmentation affects the SNV-to-gene mapping stage. SNV-to-gene mapping precedes gene scoring and gene-set analysis, and, technically, involves the generation of a file wherein each gene (one gene per row) is linked to the SNVs mapped to it (see Methods). “Augmentation” describes the addition of SNVs to a minimal SNV-to-gene mapping (referred to as the “baseline model” in our manuscript). Thus, the baseline model is always fully-contained within any augmented model. We have now clarified these points in the manuscript text, as well as with a new figure (see Fig. 1 in the revised manuscript).

Indeed, eMAGMA and H-MAGMA also modified the SNV-to-gene mapping stage of MAGMA (see Introduction). Specifically, these studies used eQTL data and Hi-C data, respectively, to assign SNVs to genes. However, a big difference with our approach was that neither study augmented a baseline model. Rather, they compared their “alternative mappings” to “default, flank-based mappings” that were not fully-contained within the former. Since the main goal of our research was to carefully scrutinize modifications to SNV-to-gene mappings, we realized that (amongst other things) it was important to have a baseline model.

**2- While interesting observations are made, many of them are not quantified (e.g., using statistical tests). It would be necessary to support observations with statistical tests. In following comments I will mention some examples, but the same idea should be applied to every observation/analysis/figure.**

We thank the referee for this comment. We now support claims made in our manuscript with appropriate statistical tests. Specifically, results from statistical tests are now provided for Fig.2, Fig.3, Fig.4, Table S3A-B, Table S4C-D, Table S5, and Table S6A-B. Note, all references are for the revised manuscript.

**3- "For instance, the fetal-brain HiC 240 dataset of RIs, outperformed most other datasets of RIs, even for non-neurological phenotypes. “ Is “outperformed” the right term? Does a larger number of genes represent a higher performance? Why? Just because more significant genes are identified, there is no guarantee that more True Positive genes are identified. In fact, in "Discussion" the authors bring up the possibility of mismatching. So maybe this should be reworded.**

We agree and now use a more careful statement (see revised manuscript, page 11, line 240, “For instance…”).

**4- This comment relates to my first comment above related to lack of description of RI augmentation: depending on how this augmentation is done, the size of RI datasets, the number of interactions for each gene, etc. may affect the number of significant genes they identify in “Gene Scoring Analyses”. The authors have evaluated one such important confounder (i.e., the coverage defined as the number of non-redundant base pairs covered by all features defining a gene, summed across all genes). This analysis is well supported by the trend in Table S2B and Figure S1A. Depending on the method of augmentation, other factors may also be important (e.g., number of interactions, etc.).**

Augmentation inevitably increases coverage, yet as the referee pointed out, it also influences many other variables. Some of these variables are known, whereas others may be unknown. Some of these variables are easily defined, whereas others are much more difficult to define (for example, consider the linkage-disequilibrium structure amongst the total collection of SNVs mapped to a gene). In addition, variables can be coupled with one another: using the referee’s example, datasets of regulatory interactions (RIs) for which there are more RIs per gene, might be associated with an increased coverage. In fact, this comment relates to our motivation for developing EPVP, which implements a control strategy in framework of MAGMA that attempts to account for all potential confounders simultaneously. As such, EPVP assigns background SNV-level associations to “extragenic SNVs” of individual genes while preserving potential confounders (like, coverage, number of RIs per gene etc.) (see Results).

**5- The caption of supplementary tables (and figures) should be expanded. They are too short and do not have sufficient information for one to understand them without checking various places in the paper, making it difficult to read.**

We have elaborated some of the captions. We hope that it reads better now.

**6- Figure 1B: Based on my understanding, each circle shows a different gene here; in addition, if more points are above the diagonal solid line, it means that larger flank has increased gene-specific scores. If that is the case, please add this description to the caption (and do the same for other figures too). Overall, the captions can be improved for all figures. Also, how about a statistical test (say one-sided Wilcoxon signed rank test) to statistically show whether there is a significant increase in the gene scores when flank size is increased from 10kb to 100kb.**

Indeed, in this figure (Fig. 2B in the revised manuscript), each gene is represented by a circle. We now clarify this in the legend. The claim we make in relation to this figure is: “To understand this trend, we studied the responses of individual genes (as opposed to genes overall) to augmentation, and established that the main effect of augmentation was to make new genes significant (that is, in most cases, comparatively few genes lost significance) (Fig. 2B and Table S3A-B).” (see revised manuscript, page 12, line 253). We now support this claim with a statistical test.

**7- Figure 1A: what is the correlation coefficient and its p-value? In general, it is necessary to quantify observations (such as this one) in the manuscript. I will not repeat my suggestions for every figure, but please add quantification in various places (it is right now lacking).**

Thank you for this comment. We have now shown Spearman’s correlation coefficients in the figure (one for each phenotype), alongside their p-values.

**8- While the permutation tests (EPVP) support that not “any” increase in the coverage would result in higher gene scores, the strong trend observed in Fig 1A and table S2B is still concerning and may suggest that this permutation test is not sufficient to rule out the possibility that size of gene region (i.g., gene body + flanks or gene body + RI regions) is the major driver in the number of significant genes. For example, if one keeps increasing the size of the flanks, well beyond 100kb, does the trend remain? At some point, we should expect that a very large flank would not be meaningful; so if the number of significant genes keeps increasing even for very large flanks, that would be concerning.**

We have now included results for gene bodies with 250kb, 500kb, and 1000kb flanks, which continue the trend (see Table S2B and Table S3B in the revised manuscript). Larger flanks were not computationally feasible. Furthermore, we now support this trend by using a second tool – Pascal (see below our response to Referee 2, comment 3).

However, we should be careful to infer anything from the results obtained for very large flanks, for two reasons. First, exceptionally large flanks might introduce confounders that do not appear with more typical SNV-to-gene mappings. Second, this experiment is not appropriate for ruling out confounding, which requires a completely random scenario (for instance, simulating GWAS data, while keeping in mind the limitations of such simulations).

Likewise, EPVP cannot rule-out confounding (since it does not reflect a *completely* random scenario). The purpose of EPVP is to have a control strategy that is compatible with MAGMA and allows us to compare genuine augmentation with matched, random augmentations. EPVP supports a contribution for SNV-level associations to the observed trend with coverage. While EPVP cannot rule-out confounding effects from augmentation, it does account for them. Thus, the purpose of EPVP is really to alleviate the “concern” that variables other than SNV-level associations, are major drivers of improvements to gene scores and gene-set scores occurring in response to augmentation (for instance, refer to the section in the Results where we validate improvements to gene-set scores).

**9- Figure 4 (and other figures): please perform statistical tests to quantify your trends and observations. E.g., in Figure 4B, you can use paired Wilcoxon signed rank test.**

We have now added a test for this Figure too (Figure 4B in the revised manuscript).

**10- Discussion: I enjoyed reading the final points about potential for mismatching. Is there other analysis you can use to test for that, since EPVP cannot? Could my suggestion above (increasing flanks sizes beyond reasonable) can (at least indirectly) provide evidence on this hypothesis?**

The main support for the mismapping hypothesis comes from the fact that (1) “reasonable” mappings (like gene bodies with small flanks) appear to identify biologically-plausible enrichments amongst gene sets and (2) “unreasonable” mappings (like gene bodies with exceptionally large flanks) do not (whilst yielding very large numbers of significant genes). This hypothesis remains speculative as long as there are no gold standards to test it. Note that we use the terminology “reasonable” here *only* to mean that gene bodies with small flanks are probably more reasonable than gene bodies with exceptionally large flanks (that is, we allude to the referee’s comment of “increasing flanks beyond reasonable”).

-------------------------------------------------------------------------------------------------------------------------------

Reviewer 2

-------------------------------------------------------------------------------------------------------------------------------

**Reviewer #2: Groenewoud et al. investigated how incorporating distal regulatory elements in gene-set based functional analysis of GWAS variants affects the numbers and identities of genes and gene-sets detected as statistically significant. They found that this approach can detect more genes than the current approach but also runs the risk of suggesting spurious associations. To overcome this issue, the authors suggest a set of additional statistical tests.**

**It is a well written paper. We give several suggestions below. As the authors will note, our main concern is a lack of clarity.**

We thank the referee for the positive feedback and the constructive criticisms.

**1. To make the paper self-contained, the concepts of competitive/comparative/non-comparative tests need more elaboration. I think, the authors might consider removing these points from the Introduction. The authors also need to clarify what they mean in the Introduction by “expression of gene score”.**

We have made appropriate changes to our manuscript text. Specifically, we have added an elaborate description of self-contained (or non-comparative) tests and competitive (or comparative) tests in the first place we refer to them (see revised manuscript, page 11, line 231, “That is…” and revised manuscript, page 16, line 336, “Unlike…”).

To improve clarity, we now avoid using the term “expression” in relation to gene scores. Thank you also for this comment.

**2. An easy-to-follow example and figure describing the gene-set analyses tools would be more helpful than the generic description starting with “Gene-set analyses surmise that the collective impact …”.**

We have included a new figure (see Fig. 1 in the revised manuscript) to explain gene-set analyses for GWAS data. We hope that it improves readability of our manuscript.

**3. Why did the authors choose MAGMA as the baseline model? This point needs a clear explanation. To what extent are the observations sensitive to the choice of MAGMA vs. other gene-set analysis models they mentioned (PASCAL and VEGAS)?**

MAGMA was our tool-of-choice (note that we reserved the term “baseline model” for something else in our manuscript) mainly because: (1) MAGMA and Pascal were developed to overcome limitations of VEGAS and (2) Pascal does not provide a way to integrate regulatory interactions (RIs) into SNV-to-gene mappings. In addition, (3) only MAGMA is still being maintained/updated regularly, (4) MAGMA is popular and user-friendly (that is, our work appeals to a significant user-base), and (5) MAGMA was likewise used for eMAGMA and H-MAGMA (publications highly-relevant to our own work).

In the revised manuscript, we show the effect of different flank sizes on the number of significant genes detected with (1) Pascal’s gene scores and (2) MAGMA’s unadjusted gene scores (that is, gene scores prior to adjustment for residual confounders, which makes them more comparable to Pascal’s gene scores) (see Table S4A-D in the revised manuscript). Importantly, the results from these two approaches are similar and consistent with our previous observations. That is, in each case, (1) larger flanks yield higher numbers of significant genes and (2) the main effect of larger flanks is to make new genes significant (that is, in most cases, comparatively few genes lose significance).

**a. The authors need to better explain MAGMA’s different steps like the “SNP-wise mean” or “adjustment for confounders” in the section titled “Gene Scoring Analyses”.**

We have now included details in the new figure (see Fig. 1 in the revised manuscript). We hope that this improves clarity.

**4. Can the authors comment to what extent the reports of e-MAGMA and H-MAGMA might be spurious (citations #40, #41)?**

We consider “spurious” to refer to improved scores obtained with augmentation that are either (1) non-specific to genuine GWAS signals (compared to permuted ones, as examined by our EPVP procedure) or (2) non-robust (as evaluated by our IRED approach). In this meaning, the term applies only to comparisons made between a baseline SNV-to-gene mapping and some augmented SNV-to-gene mapping. The studies of eMAGMA and H-MAGMA did not augment a baseline model (see also our response to Referee 1, comment 1), so we cannot directly comment on their results. However, we did augment our baseline model with datasets of RIs, which were likewise used for building the SNV-to-gene mappings of H-MAGMA (the fetal-brain and adult-brain HiC datasets, and the PsychENCODE enhancer-promoter mappings (EPMs)). We scrutinized the results obtained with the PsychENCODE EPMs using EPVP and IRED. Notably, amongst Alzheimer’s disease and schizophrenia (the two main phenotypes studied in H-MAGMA), no gene set demonstrated a robust, validated gain in response to augmentation with the PsychENCODE EPMs (see Table S11 in the revised manuscript).

**5. It is interesting how the fetal brain Hi-C RI dataset outperformed other datasets for non-neurological phenotypes. Can the authors offer any explanation? There seems to be a missed opportunity here to elaborate more on the potential caveats of “blindly” incorporating distal variants. Would other filters, such as cell/tissue-type specific accessibility help here? Or, is there an interesting similarity of regulatory mechanism between fetal brains and other organs?**

We agree that this is an interesting observation, but we now use more careful phrasing in place of “outperforms” (see Referee 1, comment 3). The simplest interpretation is that this large dataset of RIs (by coverage) (see Fig. 2A in the revised manuscript), yields many false positive genes due to mismapping. In the Discussion we elaborate on how such mismapping can take place, and indeed mention that cell-type datasets of RIs may partially resolve the issue of mismapping.

**6. Definition of “coverage” – what do the authors mean by “the features defining a gene”?**

Thank you. We have now defined this term better.

**7. The EPVP statistic is proposed to detect cases “not specific to the genuine SNV level associations, or GWAS p-values, of added SNVs”. However, the initial descriptions do not clarify how the original method takes GWAS p-values and sample-sizes into account. The EPVP statistic also needs more elaboration. Fig. 2 might be more effective with a numeric toy-example. The authors cited other papers (citations #87, #88), but the presented description is unclear in many places. Under the approach of circular shift, can it happen that the number of extragenic variants assigned to a gene actually decreases in some permutations? Due to this lack of clarity, it was not possible for this reviewer to comprehend statements like “flanks and regulatory elements to be located in parts of the genome that are generally enriched for strong phenotype associations; two variables decoupled from each other by EPVP”.**

We thank the referee for pointing out the lack of clarity in our previous description of EPVP. We have rewritten this section and hope that it reads better. Regarding the specific question raised by the referee: the permutation scheme implemented by EPVP preserves the number of SNVs assigned to each gene.

**8. The authors mention that EPVP “implied that improvement to gene scores with augmentation could occur randomly”, but did not discuss a false discovery control procedure for gene scores under augmentation approach. For gene-set analysis, however, they have shown how EPVP can invalidate certain gene sets.**

In the last section of Results (see revised manuscript, page 21, line 458, “Pinpointing genes and regulatory elements that carry the gain…”), we demonstrate the utility of EPVP to validate or invalidate gains at the gene level. See case studies in Fig. 8A-B, Fig. S7C, and Fig S8C (right panels) (note that all references are to the revised manuscript) where gene scores obtained with genuine augmentation are compared gene scores obtained (1) with the baseline model and (2) with 20 matched, random augmentations.

Given the general increase in gene scores that is typically obtained with augmentation (with genuine flanks or RIs), our study emphasizes that in order to gain informative biological insights into the etiology of the analyzed disease/trait, one should first identify significant gene-sets (based on a competitive gene-set analysis), and then seek significant genes within them, as we demonstrate in the four case studies discussed in the last section of Results.

**9. The IRED results should be presented as main figures.**

We agree. IRED is an important approach that we introduce in this study, and we have now presented some of its results as a main figure (Fig. 8).

**10. The Discussion section has a candid discussion on the potential limitations of EPVP. These points should be mentioned, at least briefly, upfront.**

We believe that EPVP is the most challenging concept introduced in our article. Therefore, to not overwhelm the reader, we decided to describe the general idea in the Results, to provide the details in the Methods, and to have a critical discussion of its advantages/disadvantages/limitations in the Discussion.

**11. Can the authors comment whether computational or experimental RIs are more likely to introduce more spurious signals?**

Datasets of RIs can differ from each other in many ways (besides whether they are based primarily on computational approaches or experimental techniques). We did not observe any clear trend. However, we did not analyze sufficient RI datasets to fully address this question.

**12. Some titles/headings are confusing: “Fig. 4. Mappings associated with more significant genes were associated with fewer significant gene sets.”**

Thank you. We have now updated some of the titles/headings for clarity.

**13. No: The authors did not make the codes available**

Upon publication, a user-friendly pipeline that executes MAGMA for a baseline model and an augmented model, alongside EPVP, will be available on GitHub: https://github.com/dgroenewoud/AUG-MAGMA
